# Sensitivity of treatment recommendations to bias in network meta‐analysis

**DOI:** 10.1111/rssa.12341

**Published:** 2017-12-06

**Authors:** David M. Phillippo, Sofia Dias, A. E. Ades, Vanessa Didelez, Nicky J. Welton

**Affiliations:** ^1^ University of Bristol UK; ^2^ Leibniz Institute for Prevention Research and Epidemiology, and University of Bremen Germany

**Keywords:** Evidence synthesis, Influence matrix, Mixed treatment comparison, Quality of evidence, Risk of bias, Threshold analysis

## Abstract

Network meta‐analysis (NMA) pools evidence on multiple treatments to estimate relative treatment effects. Included studies are typically assessed for risk of bias; however, this provides no indication of the impact of potential bias on a decision based on the NMA. We propose methods to derive bias adjustment thresholds which measure the smallest changes to the data that result in a change of treatment decision. The methods use efficient matrix operations and can be applied to explore the consequences of bias in individual studies or aggregate treatment contrasts, in both fixed and random‐effects NMA models. Complex models with multiple types of data input are handled by using an approximation to the hypothetical aggregate likelihood. The methods are illustrated with a simple NMA of thrombolytic treatments and a more complex example comparing social anxiety interventions. An accompanying R package is provided.

## 1 Introduction

Network meta‐analysis (NMA) compares the relative effectiveness of multiple treatments by combining the evidence from randomized controlled trials (RCTs), each of which compares only a subset of the treatments of interest (Lumley, 2002; Caldwell *et al*., 2005; Lu and Ades, 2006). NMA is increasingly being used by policy makers to inform treatment recommendations. However, if some of the trials included are biased then there is a risk that results from the NMA will also be biased, which could lead to suboptimal treatment recommendations.

There are numerous reasons why results from RCTs may be biased with respect to the target population for decision making, which are typically dichotomized into issues of ‘internal validity’, including poor study design or conduct, e.g. inadequate randomization or blinding, or loss to follow‐up (Schulz *et al*., 1995; Savovic *et al*., 2012a,b), and issues of ‘external validity’, affecting generalization to or representativeness of the target population (Rothwell, 2005). The potential for bias in an individual study can be assessed qualitatively by using methods such as the Cochrane risk‐of‐bias tool (Higgins *et al*., 2011). The ‘Grading of recommendations assessment, development and evaluation’ (GRADE) framework (Guyatt* et al*., 2011) can also be used to give an indication of the reliability of the evidence informing a pairwise meta‐analysis. Recently, two methods to extend the GRADE framework to NMA have been proposed (Puhan *et al*., 2014; Salanti *et al*., 2014). Although such approaches can produce valuable and necessary qualitative assessments, they cannot tell how deficiencies in internal or external validity might affect the treatment recommendation. For example, studies that are rated at high risk of bias due to issues with internal or external validity that have negligible influence on the treatment recommendation should be of little concern, whereas if they have a larger influence on the treatment recommendation then they should be scrutinized carefully.

Recently, Caldwell *et al*. (2016) proposed a method for assessing how adjustment for bias, either in individual studies or in the combined evidence on treatment contrasts, would affect the treatment recommendations from an NMA. As a form of ‘threshold analysis’, no assumptions are made regarding the source or type of bias; nor is any bias to be estimated or adjusted for; instead, thresholds are derived to show how large potential bias adjustments would need to be before the base case treatment recommendation changes, and what the new recommendation would be. The information that is provided by such a threshold analysis is therefore highly relevant to decision makers and guideline developers. Caldwell *et al*. (2016) described an iterative numerical method for obtaining bias adjustment thresholds based on a two‐stage Bayesian NMA; however, there are some limitations to this approach. Firstly, the two‐stage NMA, where pairwise meta‐analysis is performed in a first step and then each of the pairwise estimates is combined to give consistent NMA estimates, is only an approximation to the preferred one‐stage NMA where all studies on all comparisons are synthesized at once (Lu *et al*., 2011). Decision makers such as the National Institute for Health and Care Excellence recommend the one‐stage method because of its accuracy and convenience when results are used in decision models (Dias *et al*., 2013b; National Institute for Health and Care Excellence, 2013, 2014). Secondly, the numerical method is limited in its flexibility, requires the original data and full model details to be available to the analyst and can involve lengthy computation times. This paper presents an approach to threshold analysis that can be readily used by decision makers and guideline developers but avoids the limitations of the approach that was taken by Caldwell *et al*. (2016). We take a Bayesian approach to NMA because it sits naturally within a decision framework, easily extending to probabilistic cost‐effectiveness analysis (CEA) (Dias *et al*., 2013b). However, the methods apply naturally to frequentist approaches where the influence matrix is known, or (at least approximately) by setting the prior precision matrix to 0.

The remainder of this paper is structured as follows. In Section 2 bias adjustment thresholds are derived algebraically and decision invariant bias adjustment intervals are constructed, which identify precisely how large a bias adjustment can be before the recommended treatment changes. In Section 3 the method is illustrated with examples and applied to two published NMAs. Finally, results are discussed and compared with other approaches. Additional material, including detailed mathematical derivations and proofs, is provided in a Web appendix. The programs that were used to analyse the data can be obtained from

http://wileyonlinelibrary.com/journal/rss-datasets

## 2 Methods

### 2.1 Network meta‐analysis

Suppose that we have data from *n* studies on *K* treatments. Without loss of generality, treatment 1 is set as the reference against which other treatments are compared. Let *A*_*j*_ be the number of arms in study *j* ∈ {1,…,*n*}, so study *j* contributes *A*_*j*_−1 relative effects measures (data points) of the treatments in arms from 2 to *A*_*j*_, compared with that in arm 1. There are therefore $N=\Sigma_{j=1}^{n}\left(A_{j}-1\right)$ data points in total, which are contained in the data vector **y**=(*y*_1_,…,*y*_*N*_)^T^ where we assume that any multiple data entries from the same study are arranged contiguously within **y**. Each element $y_{i}:i\in \left\{1,\ldots ,N\right\}$ is a relative effect that corresponds to a comparison between treatment $t_{i}$ and comparator $c_{i}$ (also known).

We assume a multivariate normal likelihood for the data, so **y**∼*N*(***δ***,**V**) with ***δ***=(*δ*_1_,…,*δ*_*N*_)^T^ where the covariance matrix **V** is assumed known. Studies are assumed independent so that **V**=diag(**V**_1_,…,**V**_*n*_) is block diagonal, where **V**_*j*_ is the (*A*_*j*_−1)×(*A*_*j*_−1) covariance matrix for study *j*. If study *j* has only two treatments (so only one comparison is made between treatments) then **V**_*j*_ is a single element giving the variance of the corresponding relative effect.

NMA estimates basic relative treatment effect parameters *d*_*k*_, *k*=2,…,*K*, for treatment *k* compared with the reference treatment 1, with *d*_1_=0. Contrasts between any two treatments *b* and *a* can then be formed by using the consistency assumptions (Lu and Ades, 2004) as *d*_*ab*_:=*d*_*b*_−*d*_*a*_. We take a Bayesian approach, specifying prior distributions for unknown parameters and drawing conclusions from the joint posterior distribution. However, all results follow naturally for frequentist approaches, e.g. by setting the prior precision matrix $\Sigma_{d}^{-1}$ equal to **0** (see Section 4). Diffuse normal priors are usually given for the treatment effect parameters; however, all results that are presented here hold for any multivariate normal prior distribution **d**∼*N*(**d**_0_,**Σ**_*d*_), where **d**=(*d*_2_,…,*d*_*K*_)^T^.

From here, we can proceed in two ways: a fixed effect (FE) model, where all studies on a treatment contrast estimate the same treatment parameters, or a random‐effect (RE) model, where a degree of heterogeneity is allowed, and studies on a treatment contrast are assumed to estimate similar treatment parameters from a common distribution. In an FE model, $\delta_{i}=d_{t_{i}}-d_{c_{i}}$ for each *i*=1,…,*N*, which can be written concisely in matrix form as follows:

(1) $$\left.\begin{array}{cc} & \text{prior},d∼N(d_{0},\Sigma_{d}),\\ & \text{likelihood},y|d∼N(\delta ,V),\\ & \text{FE model},\delta =\mathrm{Xd}, \end{array}\right\}$$

for an appropriate *N*×(*K*−1) design matrix **X** which picks out the corresponding treatment parameters for each study contrast; for example, a study contrast comparing treatments 2 and 4 in an NMA of five treatments would have corresponding row in **X** set to (−1,0,1,0).

For an RE model with two‐arm trials, $\delta_{i}∼N\left(d_{t_{i}}-d_{c_{i}},\tau^{2}\right)$ for each *i*=1,…,*N*. For simplicity of exposition, we assume that the between‐study variance $\tau^{2}$ is homogeneous between all treatment contrasts; however, the derivations are identical with distinct between‐study variances for each contrast. The FE model can be thought of as a special case of the RE model, where the between‐study variance $\tau^{2}$ is set to 0.

If there are trials with more than two arms, then a multivariate normal distribution is required to capture the correlations between the estimated relative effects from the same RCT (Higgins and Whitehead, 1996). In general, the RE model can be written as follows:

(2) $$\left.\begin{array}{cc} & \text{prior},d∼N(d_{0},\Sigma_{d}),\tau ∼\pi ,\\ & \text{likelihood},y|\delta ∼N(\delta ,V),\\ & \text{RE model},\delta |d,\tau^{2}∼N\left(\mathrm{Xd},\Sigma_{\tau^{2}}\right), \end{array}\right\}$$

with some prior *π* on *τ* (or $\tau^{2}$) such as *τ*∼*U*(0,10), and where the between‐studies covariance matrix $\Sigma_{\tau^{2}}$ is of the form **A** *τ* ^2^ where **A** is a block diagonal ‘design matrix’. Since here $\tau^{2}$ is assumed to be the same between all contrasts, the block of **A** corresponding to a study reporting relative effects with three or more arms will have 1s on the diagonal and 0.5s everywhere else (Higgins and Whitehead, 1996). Note that the between‐study variance is assumed homogeneous for simplicity only; the derivations proceed identically with a generic covariance matrix $\Sigma_{\tau^{2}}$.

Although we have considered only data in relative effects form here, all results apply easily to data in absolute effects (arm level) form (or even mixtures of the two) simply by modifying the design and covariance matrices appropriately.

### 2.2 Decision rule

We assume that the decision is made on the basis of the estimated relative treatment effects from the joint posterior distribution of *d*_2_,…,*d*_*K*_, and (without loss of generality) we assume that a larger observed outcome (e.g. log‐odds of success) is preferable. The optimal treatment is chosen to be that which has the highest expected treatment effect, i.e. $k^{*}$ which satisfies $E_{d|y}\left(d_{k^{*}}\right)⩾E_{d|y}\left(d_{k}\right),\forall \;k=1,\ldots ,K$. For brevity, we write E(·) in place of $E_{d}{|}_{y}\left(\cdot \right)$, so that

(3) $$k^{*}:=\underset{k=1,\ldots ,K}{\text{arg max}}E\left(d_{k}\right).$$

Note that other decision rules could be considered, e.g. a rule based on a minimally important difference, or maximizing the expected net benefit from an economic model (see Section 4).

### 2.3 Deriving bias adjustment thresholds at study level

We begin by considering bias adjustments to individual study estimates of treatment effect one at a time, i.e. for each data point *y*_*m*_. The methods that are described in this section and Section 2.4 below are repeated for each *m* ∈ {1,…,*N*} separately. In Section 2.5 we extend the methods to consider bias adjustments for multiple data points.

Suppose that some study data point *y*_*m*_, instead of estimating the true value of $d_{t_{m}c_{m}}$, is biased so that it estimates $d_{t_{m}c_{m}}-\beta_{m}$. We aim to find threshold values for *β*_*m*_ at which the overall decision based on equation (3) changes. For this we consider hypothetical data that have been bias adjusted, $\tilde{y}$, on which we could perform the NMA to obtain the ‘true’ treatment effect. We define the bias‐adjusted data as $\tilde{y}\left(\beta_{m}\right)=y+\beta_{m}$, where the *i*th component of the vector ***β***_*m*_ is

(4) $${\left[\beta_{m}\right]}_{i}=\left\{\begin{array}{cc} \beta_{m} & \;\text{if}\;i=m\\ 0 & \;\text{if}\;i\neq m. \end{array}\right.\forall \;\;i\in \left\{1,\ldots ,N\right\}$$

We shall denote posterior expectation with respect to the bias‐adjusted data by $\tilde{E}\left(\cdot \right):=E_{d|\tilde{y}\left(\beta_{m}\right)}\left(\cdot \right).$

#### 2.3.1 General form of bias adjustment thresholds

We wish to find the smallest positive and negative values of the bias adjustment such that the optimal treatment $k^{*}$ given by equation (3) changes; we call these values *bias adjustment thresholds* and denote them $\beta_{m}^{+\mathrm{thresh}}$ and $\beta_{m}^{-\mathrm{thresh}}$ respectively. At each threshold value there is a new treatment ${\tilde{k}}^{*}$ that achieves the maximum posterior expected treatment effect.

To find the threshold values, we consider a set of *K*−1 possible solutions $\left\{u_{ak^{*},m}:a\in \left\{1,\ldots ,K\right\}\backslash k^{*}\right\}$, where each $u_{ak^{*},m}$ reflects the amount of bias adjustment to data point *y*_*m*_ required to change the sign of $E(d_{ak^{*}})$ and to make treatment *a* more efficacious in expectation than the current optimal treatment $k^{*}$. The threshold values $\beta_{m}^{+\mathrm{thresh}}$ and $\beta_{m}^{-\mathrm{thresh}}$ are simply the smallest positive and negative solutions from this set:

(5) $$\begin{array}{cc} \beta_{m}^{+\mathrm{thresh}}=\; & u_{bk^{*},m}b=\underset{a\in \left\{1,\ldots ,K\right\}\backslash k^{*}}{\arg\min}\left\{u_{ak^{*},m}:u_{ak^{*},m}>0\right\},\\ \beta_{m}^{-\mathrm{thresh}}=\; & u_{bk^{*},m}b=\underset{a\in \left\{1,\ldots ,K\right\}\backslash k^{*}}{\arg\max}\left\{u_{ak^{*},m}:u_{ak^{*},m}<0\right\}. \end{array}$$

The possible solutions $u_{ak^{*},m}$ are determined by the expected difference in treatment effects, $-E(d_{ak^{*}})$, divided by the amount of influence that *y*_*m*_ has on the expected difference, given by a linear combination of elements of the influence matrix $H:\;\text{for}\;k^{*}\neq 1$

(6a) $$\begin{array}{cc} & u_{ak^{*},m}=\frac{-E(d_{ak^{*}})}{{\left[H\right]}_{k^{*}-1,m}-{\left[H\right]}_{a-1,m}},\text{for}\;a\in \left\{2,\ldots ,K\right\}\backslash k^{*},\\ & u_{1k^{*},m}=\frac{-E(d_{1k^{*}})}{{\left[H\right]}_{k^{*}-1,m}},\text{for}\;a=1; \end{array}$$

for $k^{*}=1$

(6b) $$u_{1a,m}=\frac{-E(d_{1a})}{{\left[H\right]}_{a-1,m}},\text{for}\;a\in \left\{2,\ldots ,K\right\}.$$

[**H**]_*a*−1,*m*_ is the entry in the (*a*−1)th row and *m*th column of **H**, the influence matrix that maps the data **y** onto the posterior estimates of the basic treatment effect parameters **d**:

(7) $$\tilde{E}\left(d\right)=E\left(d\right)+H\beta$$

for any general vector ***β*** changing the observed data **y** to bias‐adjusted data $\tilde{y}\left(\beta \right)=y+\beta$. The exact form of **H** will depend on the model and is described in the following sections for some typical NMA models. The influence matrix is related to the hat matrix (Konig *et al*., 2013; Krahn *et al*., 2013; Salanti *et al*., 2014); see Section 4.

The new optimal treatment at the thresholds could be found by using equation (3), which requires re‐evaluating the joint posterior mean and taking a maximum for each $\beta_{m}^{+\mathrm{thresh}}$ and $\beta_{m}^{-\mathrm{thresh}}$. However, lemma 1 (in on‐line Appendix A.1) shows that a more efficient approach is simply to note the new optimal treatment from the contrast whose posterior expectation changes sign at the bias adjustment threshold—treatment *b* from equation (5).

**Figure 1:**
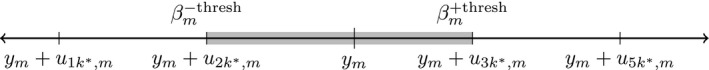
Example construction of a decision invariant bias adjustment interval (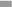) for a data point *y*_*m*_, for an NMA with five treatments and current optimal treatment $k^{*}\text{=}\;4$: the new treatment decision at the negative and positive thresholds would be 2 and 3 respectively

From the positive and negative bias adjustment thresholds, it is intuitive to think of constructing an interval $\left(y_{m}+\beta_{m}^{-\mathrm{thresh}},y_{m}+\beta_{m}^{+\mathrm{thresh}}\right)$ within which a bias‐adjusted value of ${\tilde{y}}_{m}$ can lie without changing the treatment decision. We refer to such an interval as the *decision invariant bias adjustment interval* about *y*_*m*_ and visualize this as shown in Fig. 1.

Thresholds and invariant intervals may be derived for more complex treatment decisions, as well as the simple ‘maximal efficacy’ decisions that were described above. For example, decision makers may be interested in the level of bias adjustment that would be required to make another treatment significantly more effective than the base case optimal treatment, as judged by some minimal clinically important difference *ρ*. In this case, the thresholds are found in the usual manner (equation (5)) from the set of possible solutions now given as follows: $\text{for}\;k^{*}\neq 1$

$$\begin{array}{cc} & u_{ak^{*},m}=\frac{-E(d_{ak^{*}})-\rho }{{\left[H\right]}_{k^{*}-1,m}-{\left[H\right]}_{a-1,m}},\text{for}\;a\in \left\{2,\ldots ,K\right\}\backslash k^{*},\\ & u_{1k^{*},m}=\frac{-E(d_{1k^{*}})-\rho }{{\left[H\right]}_{k^{*}-1,m}},\text{for}\;a=1; \end{array}$$

for $k^{*}=1$

$$u_{1a,m}=\frac{-E(d_{1a})+\rho }{{\left[H\right]}_{a-1,m}},\text{for}\;a\in \left\{2,\ldots ,K\right\}.$$

More complex threshold analyses, e.g. for specific biases, may be undertaken by examining the set of *u*_*ab*,*m*_ values from equation (6) (for an example, see Section 3.2.2).

#### 2.3.2 Bias adjustment thresholds for the fixed effect model

For the FE model with conjugate normal prior distribution for the treatment effect parameters **d** (equation (1)), the on‐line appendix A.2 (see also Gelman *et al*. (2013), page 71) shows that the posterior distribution is

(8) $$d|y∼N\{\Sigma_{n}\left(\Sigma_{d}^{-1}d_{0}+X^{T}V^{-1}y\right),\Sigma_{n}\}$$

where the posterior covariance matrix is $\Sigma_{n}={\left(\Sigma_{d}^{-1}+X^{T}V^{-1}X\right)}^{-1}$.

The threshold values are found by using equations (5) and (6b), where the influence matrix is **H**=**Σ**_*n*_ **X** ^T^ **V** ^−1^ (on‐line appendix A.3).

#### 2.3.3 Bias adjustment thresholds for the random‐effects model

The RE model (equation (2)) is typically specified with a prior distribution over the between‐studies standard deviation *τ* which, because of the hierarchical nature of the model, results in a joint posterior distribution that generally has no closed form solution. One approach in this situation would be to find bias adjustment thresholds numerically by iteratively changing the data until the decision changes; this is likely to be very computationally expensive. However, approximate *algebraic* bias adjustment thresholds can be obtained for the RE model by considering the between‐studies variance to be known, fixed and unchanged after bias adjustment. Sensitivity analyses may then be performed to assess how the thresholds change for various values of $\tau^{2}$.

For the RE model given in equation (2) with $\tau^{2}$ assumed known and fixed, the on‐line appendix A.4 (see also Gelman *et al*. (2013), page 582) shows that the joint posterior distribution for **d** and ***δ*** is

(9) $$\begin{array}{cc} & \left(\begin{array}{c} d\\ \delta \end{array}\right)|y,\tau^{2}∼N\left\{\Sigma_{n}\left(\begin{array}{c} \Sigma_{d}^{-1}d_{0}\\ V^{-1}y \end{array}\right),\Sigma_{n}\right\},\\ & \Sigma_{n}={\left(\begin{array}{cc} X^{T}\Sigma_{\tau^{2}}^{-1}X+\Sigma_{d}^{-1} & -X^{T}\Sigma_{\tau^{2}}^{-1}\\ -\Sigma_{\tau^{2}}^{-1}X & V^{-1}+\Sigma_{\tau^{2}}^{-1} \end{array}\right)}^{-1}=\left(\begin{array}{cc} A_{*} & B_{*}\\ B_{*}^{T} & C_{*} \end{array}\right), \end{array}$$

where the posterior covariance matrix **Σ**_*n*_ is partitioned according to the dimensions of **d** and ***δ***. Under bias‐adjusted data, it can be shown (on‐line appendix A.5) that the joint posterior mean becomes $\tilde{E}\left(d\right)=E\left(d\right)+B_{*}V^{-1}\beta_{m}$. Following the same arguments as for the basic FE case (on‐line appendix A.3), the thresholds are given by equations (5) and (6b) where the influence matrix is now **H**=B_*_ **V** ^−1^.

The posterior covariance matrix **Σ**_*n*_ is the inverse of a block matrix and so can be calculated explicitly (see Bernstein (2005), page 45); it is, however, more likely that Σ_*n*_ will have been estimated by using Bayesian software such as WinBUGS (Lunn *et al*., 2000). We can then simply partition the posterior covariance matrix as in equation (9) to obtain B_*_.

#### 2.3.4 Extended models with additional parameters

We may wish to add additional parameters to the basic FE and RE models (Sections 2.3.2 and 2.3.3), e.g. to include data as absolute effect measures (i.e. as one observation per study arm) where a nuisance study level baseline parameter for arm 1 is included (Lu and Ades, 2006). We denote the additional parameters by ***μ*** and give them a normal prior distribution ***μ***∼*N*(***μ***_0_,Σ_***μ***_).

The simplest way to achieve this for the FE model is to extend the parameter vector to $\gamma =(\begin{array}{c} d\\ \mu \end{array})$. The design matrix **X** is also extended to describe the model. The on‐line appendix A.6 shows that we obtain the threshold equations (5) and (6b), where the influence matrix is now **H**=[**Σ**_*n*_ **X** ^T^ **V** ^−1^]_rows 1:*K*−1_.

For the RE model, the additional parameters have an associated design matrix **M**, and we impart further flexibility with a design matrix **L** for ***δ***. The on‐line appendix A.7 shows that the thresholds are given by equations (5) and (6b) with **H**=(B_*_ **L** ^T^+D_*_ **M** ^T^)**V** ^−1^. Here, analogously to equation (9), B_*_ and D_*_ are partitions of the posterior covariance matrix, corresponding to the covariance of **d** with ***δ*** and of **d** with ***μ*** respectively.

#### 2.3.5 Class effect random‐effect model

Class effect models are often utilized in NMAs where treatment effects may be assumed exchangeable within discrete classes, e.g. based on common constituent compounds or modes of action (Dominici *et al*., 1999; Mayo‐Wilson *et al*., 2014). In such models, treatment effects within the same class are assumed exchangeable and normally distributed as $d|z∼N(\mathrm{Zz},\Sigma_{d})$, with class effect parameters **z** and class design matrix **Z** assigning a class to each treatment. The class effect parameters are given a normal prior distribution. **Σ**_*d*_ is the between‐treatment covariance matrix, which may specify a common within‐class variance or different within‐class variances for each class. If **Σ**_*d*_ is the zero matrix then the model is equivalent to fixed class effects.

To proceed analytically we assume that the between‐studies variance $\tau^{2}$ is fixed, known and invariant to bias adjustment (as with the RE model in Sections 2.3.3 and 2.3.4); we must also make the same assumptions about the within‐class variances (for the random class effect model). In the on‐line appendix A.8 we show that the influence matrix for an RE model including class effects is identical to that in the extended RE case in Section 2.3.4; we may proceed exactly as in the extended RE case despite the presence of class effects.

### 2.4 Bias adjustment thresholds at the contrast level

In clinical guideline development, assessment of the quality of evidence is often directed at the entire body of evidence on a contrast rather than at individual studies. This is the method of evidence classification that is used in, for example, extensions of GRADE to NMA (Puhan *et al*., 2014; Salanti *et al*., 2014). We may therefore wish to examine the robustness of treatment decisions to bias in the combined body of evidence at contrast level, rather than for individual studies. In some cases it may only be possible to obtain decision invariant thresholds at the contrast level, i.e. when only the summary results (posterior means and covariance matrix for all parameters) from an NMA are available. Alternatively the NMA may entail a complex, hierarchical or otherwise analytically intractable model but where the joint posterior distribution for the treatment effect parameters can be assumed to be approximately multivariate normal.

Our approach is to consider a hypothetical data set, consisting of a single independent data point for each contrast where there is direct evidence, which when pooled by using an FE NMA gives a posterior distribution that closely approximates the true posterior distribution as reported by the original NMA. We are not suggesting independence of the original data, but that the posterior distribution could have arisen (at least approximately) from an alternative set of independent data points. Multiarm trials, REs and other features are therefore handled as usual in the original NMA, and all correlations and uncertainty appropriately propagated into the joint posterior distribution on which the contrast level threshold analysis is based. We show that, to derive thresholds, we need only the covariance matrix of the hypothetical data, and not the hypothetical data points themselves. We then proceed to derive thresholds as for the basic FE model that was described in Section 2.3.2.

We consider a hypothetical data set consisting of single independent data points *y*_*ab*_ with variances *v*_*ab*_, representing the combined evidence on each contrast *d*_*ab*_ where there is direct evidence, with multivariate normal likelihood **y**|**d**∼*N*(**Xd**,**V**) where **X** is a design matrix and **V** is diagonal with elements *v*_*ab*_. We design the hypothetical data set so that pooling using an FE NMA gives a posterior distribution $N(\hat{\eta },\hat{\Sigma })$ that closely approximates the true posterior distribution *N*(***η***,**Σ**) that is reported by the original NMA. Thresholds can then be derived as for the basic FE model in Section 2.3.2.

A full derivation of the contrast level method is given in the on‐line appendix A.9. We choose **V** to solve $\Sigma =\hat{\Sigma }$, where the covariance matrix of the reconstructed posterior distribution is $\hat{\Sigma }={\left(\Sigma_{d}^{-1}+X^{T}V^{-1}X\right)}^{-1}$ (see Section 2.3.2). When the evidence network is complete (i.e. every treatment is joined to every other by direct evidence), there is a unique exact solution; otherwise an approximate solution is found by using non‐negative least squares (Lawson and Hanson, 1995). In the latter case, the performance of the approximation may be assessed by examining the Kullback–Leibler divergence (Kullback and Leibler, 1951) of the reconstructed posterior distribution from the true posterior distribution. Interpreting the Kullback–Leibler divergence as a log‐Bayes‐factor, values less than 1 indicate negligible differences between the reconstructed posterior from the true posterior and a good approximation, whereas values greater than 3 indicate considerable differences and a poor approximation (Kass and Raftery, 1995).

Once the hypothetical likelihood covariance matrix has been reconstructed, the thresholds are then evaluated as before by using equations (5) and (6b) with the influence matrix **H**=**ΣX** ^T^ **V** ^−1^. Note that it would not be possible to re‐evaluate the posterior means under the bias‐adjusted data to obtain ${\tilde{k}}^{*}$ as we do not have the hypothetical data, but we can use the result of lemma 1 (on‐line appendix A.1) to obtain the new optimal treatment efficiently, as before.

### 2.5 Thresholds for bias in multiple studies or contrasts

**Figure 2:**
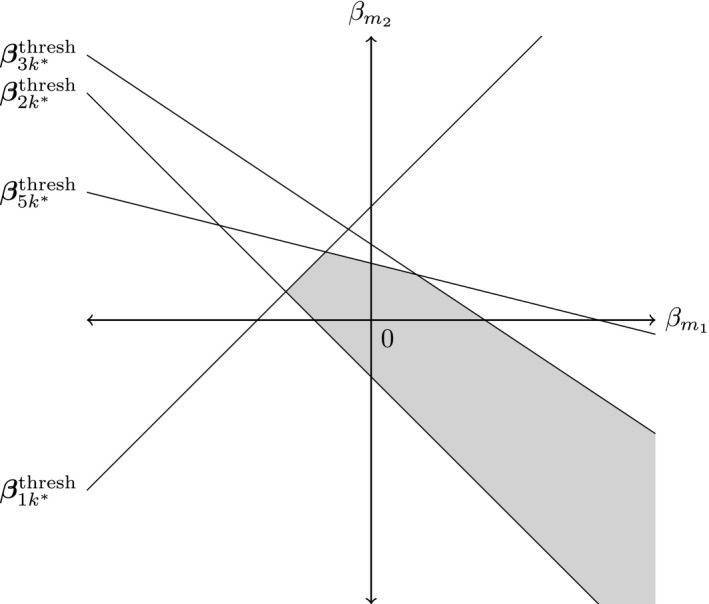
Example of thresholds lines in two dimensions for an NMA of five treatments with *k* ^*^=4 (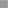, invariant region about the origin (no bias adjustment)): any simultaneous bias adjustment $\left(\beta_{m_{1}},\beta_{m_{2}}\right)$ to data points $y_{m_{1}}$ and $y_{m_{2}}$ which remains within the invariant region does not change the optimal treatment; at the boundary of the invariant region formed by thresholds line $\beta_{ak^{*}}^{\mathrm{thresh}}$ the new optimal treatment is $\tilde{k}{}^{*}=a$

Thus far we have been concerned with the effects of bias adjustment for a single data point at a time. However, we may wish to consider the effect of bias adjustment in multiple studies or contrasts simultaneously, e.g. all the relative effects estimates from a multiarm study, or perhaps multiple studies that are of concern. Such analyses are possible at both study and contrast level, though they are more likely to be motivated by knowledge of individual trials and their characteristics. Equation (7) shows how a general bias adjustment ***β*** would change the posterior mean of the treatment effect parameters. We extend the approach that is taken in the on‐line appendix A.3 to let two elements of ***β*** be non‐zero in equation (4), allowing for bias adjustment in two data points $y_{m_{1}}$ and $y_{m_{2}}$ simultaneously. We end up solving *K*−1 equations in two unknowns:

(10) $$\begin{array}{cc} & 0=\tilde{E}\left(d_{ak^{*}}\right)=E\left(d_{ak^{*}}\right)+\left({\left[H\right]}_{k^{*}-1,m_{1}}-{\left[H\right]}_{a-1,m_{1}}\right)\beta_{m_{1}}+\left({\left[H\right]}_{k^{*}-1,m_{2}}-{\left[H\right]}_{a-1,m_{2}}\right)\beta_{m_{2}},\\ & \forall \;a\in \left\{1,\ldots ,K\right\}\backslash k^{*} \end{array}$$

where $m_{1}$ and $m_{2}$ are the indices of the two data points to be bias adjusted. We see that, instead of threshold points, we have *K*−1 threshold lines in two dimensions. By rearranging equation (10) and using the definition of $u_{ak^{*},m}$ from equation (6), we can use the set of previously calculated $u_{ak^{*},m}$ to arrive at the equation for each threshold line $\beta_{ak^{*}}^{\mathrm{thresh}}$: $\beta_{m_{2}}=u_{ak^{*},m_{2}}-\left(u_{ak^{*},m_{2}}/u_{ak^{*},m_{1}}\right)\beta_{m_{1}}$. The intersection of these threshold lines creates a bias invariant region, e.g. as portrayed in Fig. 2 for an NMA of five treatments where the current optimal treatment is $k^{*}=4$.

It is simple both mathematically and computationally to carry on extending such a technique to higher dimensions: allowing *r*⩽*N* components of ***β*** to be non‐zero results in *r*‐dimensional threshold hyperplanes $\beta_{ak^{*}}^{\mathrm{thresh}}$ with equations $\beta^{T}w_{ak^{*}}=1$ for $a\in \left\{1,\ldots ,K\right\}\backslash k^{*}$, where the *i*th component of $w_{ak^{*}}$ is

$${\left[w_{ak^{*}}\right]}_{i}=\left\{\begin{array}{cc} u_{ak^{*},i}^{-1} & \text{if}\;i\in \{m_{1},\ldots ,m_{r}\}\\ 0 & \text{otherwise}. \end{array}\right.\forall \;i\in \left\{1,\ldots ,N\right\}$$

However, beyond two (or possibly three) dimensions it becomes impossible to visualize and analyse these threshold hyperplanes effectively and the resulting invariant hypervolume formed by their intersection. As such, any analysis of simultaneous bias adjustment is probably best approached in a targeted manner, identifying a small number of data points on which to focus attention.

An alternative approach is to report the vectors $\beta_{ak^{*}}^{\min}=w_{ak^{*}}/{\left\|w_{ak^{*}}\right\|}^{2}$ for $a\in \left\{1,\ldots ,K\right\}\backslash k^{*}$ giving the point on each threshold hyperplane $\beta_{ak^{*}}^{\mathrm{thresh}}$ which lies closest to the origin and so minimizes the amount of overall bias adjustment required to change the optimal treatment decision to ${\tilde{k}}^{*}=a$.

## 3 Examples

We apply the threshold method to two examples: firstly, an NMA of thrombolytic treatments (Caldwell *et al*., 2005) to demonstrate study and contrast level analyses on a simple FE model, along with simultaneous bias adjustment in two data points; secondly, a large class effects RE NMA comparing treatments for social anxiety (National Collaborating Centre for Mental Health, 2013; Mayo‐Wilson *et al*., 2014) to demonstrate the power of a contrast level analysis when applied to complex models. Notes on practical computation are included in the on‐line appendix A.10. Code is provided at http://wileyonlinelibrary.com/journal/rss-datasets along with an R package implementing the threshold method for general use in the on‐line supplementary materials.

### 3.1 Example: thrombolytics

**Figure 3:**
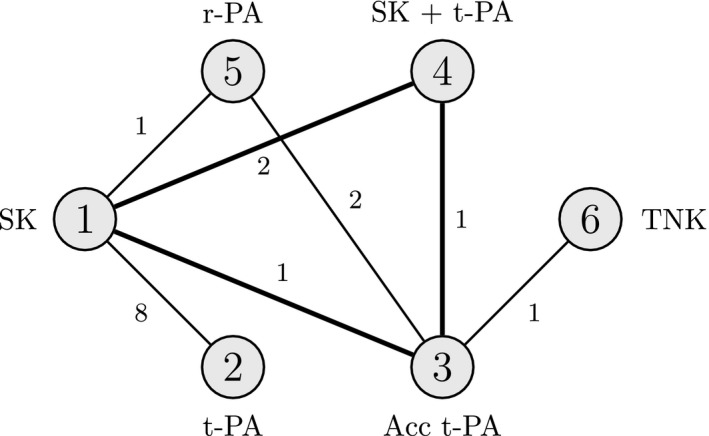
Thrombolytics example network, showing how the six treatments are connected by study evidence: nodes represent treatments and edges show comparisons made by studies; numbers inside the nodes are the treatment codings; numbers on the edges give the number of studies making that comparison; the bold triangle is the loop formed by the three‐arm study

Fig. 3 shows the network of treatment comparisons for *K*=6 thrombolytic treatments based on *n*=14 studies, taken from two systematic reviews (Boland *et al*., 2003; Keeley *et al*., 2003). Previous work has shown that an FE model is appropriate for the data (Caldwell *et al*., 2005; Dias, Welton, Sutton, Caldwell, Lu and Ades, 2013). All studies have two arms apart from one with three, so the number of data points (log‐odds‐ratios (ORs)) is *N*=15.

#### 3.1.1 Study level fixed effects analysis

An FE model was fitted to the data by using WinBUGS 1.4.3 (Lunn *et al*., 2000) and code from Dias, Welton, Sutton, Caldwell, Lu and Ades (2013). The treatment effect parameters *d*_*k*_ are interpreted as the log‐OR of mortality between treatment *k* and the reference treatment 1, and *d*_1_=0. In this example the optimum treatment is the one which minimizes the log‐OR of mortality, here $k^{*}={\arg\min}_{k=1,\ldots ,6}E\left(d_{k}\right)=3$ (full results of the NMA are available in the accompanying R package).

**Figure 4:**
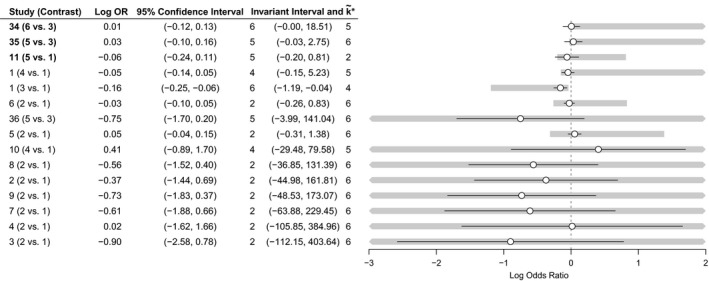
Study level forest plot, displaying invariant intervals for the thrombolytics example, sorted with smallest thresholds first (bold labels in the table emphasize study estimates with short invariant intervals lying within the 95% CI; the optimal treatment without bias adjustment is k*=3): ∘, log‐OR;—, 95% CI; 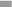, invariant interval

The results of each study and the study level threshold analysis are shown in Fig. 4: the table on the left‐hand side displays the estimated log‐OR from each study comparison along with a 95% confidence interval (CI), and a decision invariant bias adjustment interval about the estimate, showing how far any bias adjustment can be made before the optimal treatment changes. The new optimal treatments ${\tilde{k}}^{*}$ are reported alongside either end of the invariant interval. The right‐hand side of Fig. 4 displays these graphically, with points and lines for estimated log‐ORs and their CIs and shaded bands for the invariant regions. Where a 95% CI extends beyond the invariant interval the study label is bold, indicating that the treatment recommendation is sensitive to the level of imprecision in this study estimate. In this example, the treatment recommendation is sensitive to the level of imprecision in studies 11 and 35 (${\tilde{k}}^{*}=5$) and study 34 (${\tilde{k}}^{*}=6$). For example, the estimated log‐OR of 0.01 for treatment 6 *versus* 3 in study 34 has an invariant interval of (−0.00, 18.51); a change to the log‐OR of only −0.01 in favour of treatment 6 (either due to bias adjustment, or simply random sampling error) is enough to change the optimal treatment from $k^{*}=3$ to ${\tilde{k}}^{*}=6$. Looking at the network of treatments (Fig. 3), this sensitivity is not surprising. Treatment 6 is only compared directly with treatment 3, and only in study 34, which found no evidence of a significant difference between the two treatments (the 95% CI for the log‐OR contains zero). Since $k^{*}=3$, adjusting the log‐OR to be in favour of treatment 6 means that the network of evidence behind treatment 3 now points to ${\tilde{k}}^{*}=6$. Similarly, changes of −0.06 and −0.14 to the treatments 5 *versus* 3 and 5 *versus* 1 comparisons of studies 35 and 11 respectively both result in ${\tilde{k}}^{*}=5$ becoming optimal. Other studies may be biased beyond the range of the 95% CI; our method highlights that the treatment recommendation may be sensitive to plausible bias adjustments in studies 1, 5 and 6, each with thresholds less than 0.5 on the log‐OR scale. All studies with plausibly small thresholds should be assessed for risk of bias, e.g. by using GRADE (Guyatt *et al*., 2011) or the Cochrane risk‐of‐bias tool (Higgins *et al*., 2011), and the thresholds and invariant intervals interpreted in light of the expected magnitude and direction of bias, e.g. novelty bias favouring a new treatment. The metaepidemiological literature on the empirical evidence for bias is likely to help to determine plausible magnitudes for biases (Savovic *et al*., 2012a). In the remaining studies, our method reveals that no changes—no matter how large—can ever plausibly lead to changes in the treatment recommendation, particularly studies 2–4 and 7–10, since the bias adjustment thresholds are infeasibly large on the log‐OR scale.

#### 3.1.2 Contrast level analysis

We also perform a contrast level analysis to examine sensitivity to changes in the aggregate bodies of evidence on each contrast. We do not need the original data to do this; we use only the posterior means and covariance matrix from the joint posterior distribution of the treatment effect parameters **d**=(*d*_2_,…,*d*_6_)^T^. We treat the posterior distribution as if it arose from an NMA on seven independent data points **y**=(*y*_12_,*y*_13_,*y*_14_,*y*_15_,*y*_34_,*y*_35_,*y*_36_)^T^—the number of direct comparisons in the treatment network (i.e. the edges in Fig. 3). Each data point *y*_*ab*_ represents the combined direct study evidence on a treatment contrast *d*_*ab*_.

Following the methods in Section 2.4, we construct an approximate hypothetical likelihood by using non‐negative least squares—see the on‐line appendix A.11. The Kullback–Leibler divergence of the reconstructed posterior distribution from the true posterior distribution is very small at 6.76×10^−5^, indicating that the hypothetical data are a good approximation.

**Figure 5:**
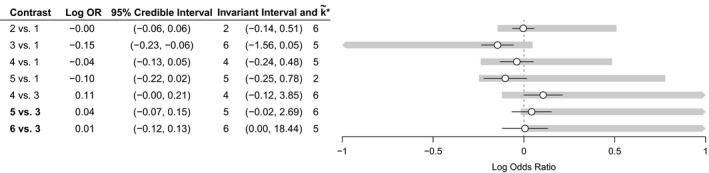
Contrast level forest plot, displaying invariant intervals for the thrombolytics example (bold labels in the table emphasize contrast estimates with short invariant intervals lying within the 95% credible interval; the optimal treatment without bias adjustment is k*=3): ∘, log‐OR;—, 95% credible interval; 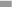, invariant interval

Fig. 5 presents results of the NMA and the contrast level analysis, which echoes the study level analysis. Notably the thresholds for contrasts where there is a single two‐arm study making the comparison (treatments 6 *versus* 3 and 5 *versus* 1) match almost exactly with the thresholds for the corresponding studies in the study level analysis (Section 3.1.1), as expected. Furthermore, we clearly see the effects of bias adjustment on entire bodies of evidence in comparison with the study level approach: individually, studies making the treatments 2 *versus* 1 comparison have little influence on the treatment decision, shown by wide invariant intervals (Fig. 4); when the evidence from these studies is considered collectively for bias adjustment, the combined invariant interval becomes narrower. Indeed, a combined bias adjustment of −0.14 in favour of treatment 2 may be plausible. Note here that the black lines in Fig. 5 correspond to the 95% credible intervals for each contrast estimate resulting from the NMA, instead of CIs for each study estimate as in the study level analysis (Fig. 4).

The threshold analysis gives very small thresholds for the combined evidence on treatment contrasts 5 *versus* 3 and 6 *versus* 3, which is symptomatic of the lack of evidence for significant differences between these treatments. A likely treatment decision in such a scenario (in the absence of issues surrounding cost or adverse events) would therefore be to recommend any of these three treatments.

#### 3.1.3 Simultaneous bias adjustment in two data points

**Figure 6:**
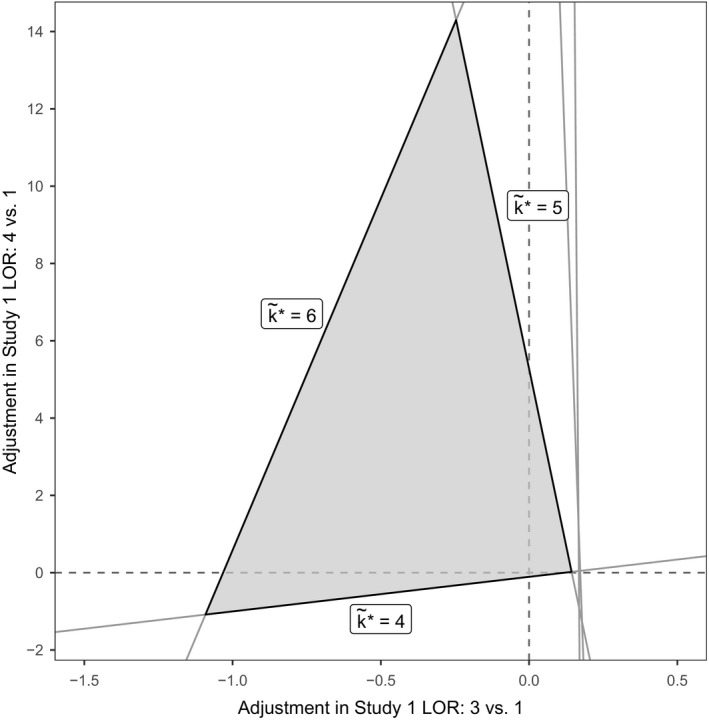
Invariant region formed from threshold lines for bias adjustment to the two relative effect estimates from study 1 (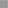, invariant region): the new optimal treatments on the boundary are indicated by ${\tilde{k}}^{*}$; optimal treatment without bias adjustment is *k* ^*^=3

We shall now consider analysing bias adjustment in two data points simultaneously. Such an analysis is possible at both study and contrast level, though it is more likely to be motivated by knowledge of individual trials and their characteristics; thus we shall return to the study level scenario for this example. In the thrombolytics data set, study 1 was a three‐armed study comparing treatments 1, 3 and 4, resulting in two log‐OR estimates against the reference treatment 1. The two log‐ORs are not independent, and so if bias adjustment is required it is possible that both estimates will need to be bias adjusted together—if the trial failed to blind patients, for example. Fig. 6 presents the invariant region for simultaneous bias adjustments in the two log‐ORs estimated by study 1, formed by the polygon of intersecting threshold lines about the origin, which can either be closed (threshold lines in every direction) or open (bias adjustment in some direction will never cross a threshold line). In this example, three threshold lines form an invariant region for bias adjustment, with new optimal treatments at the thresholds $k^{*}=4,5,6$. The points where the boundaries of the invariant region intersect the axes correspond to the one‐dimensional invariant intervals that were presented in Fig. 4, since setting one of the two bias adjustments to 0 returns us to analysing a bias adjustment in one data point only. Of particular interest are the threshold lines for ${\tilde{k}}^{*}=4$ and ${\tilde{k}}^{*}=5$ which lie closest to the origin. In the one‐dimensional case we saw that, individually, bias adjustments of 0.12 in the log‐OR of treatment 3 *versus* 1 or −0.11 in the log‐OR of treatment 4 *versus* 1 were needed to change the optimal treatment to ${\tilde{k}}^{*}=4$, and a bias adjustment of 5.28 in the log‐OR of treatment 4 *versus* 1 was needed to change the optimal treatment to ${\tilde{k}}^{*}=5$ (Fig. 4). Now, allowing both estimates to be bias adjusted simultaneously, we see that it is possible to arrive at ${\tilde{k}}^{*}=5$ with much smaller amounts of bias adjustment than this; for example with bias adjustments of just 0.14 to the treatment 3 *versus* 1 log‐OR and 0.02 to the treatment 4 *versus* 1 log‐OR we cross the invariant threshold and would recommend treatment 5.

### 3.2 Example: social anxiety

**Figure 7:**
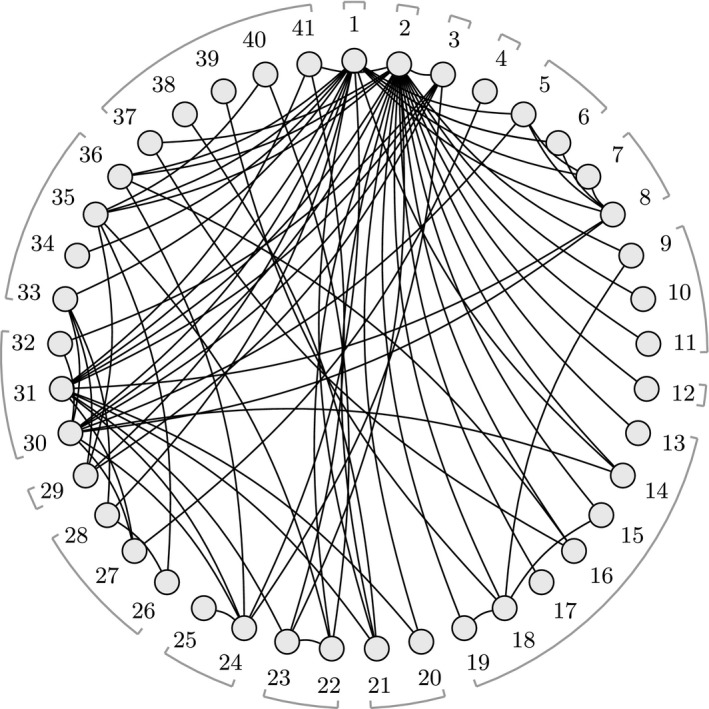
Social anxiety treatment network: nodes represent treatments and edges show study comparisons; numbers around the edge are the treatment codings; treatment classes are indicated by the braces (some classes contain a single treatment only); treatment 1 is waitlist, treatment 2 is pill placebo and treatment 3 is psychological placebo; Table A1 in the on‐line appendix A.12 lists the treatment codes and classes

We now consider a more complex example where analysis is greatly simplified by using the contrast level approach. Fig. 7 shows the network for an NMA of 41 interventions for social anxiety from 100 studies (National Collaborating Centre for Mental Health, 2013; Mayo‐Wilson *et al*., 2014). The original analysis uses an RE model which includes class effects for 17 different treatment classes and a secondary network of studies for a regression calibration on recovery. No single common outcome measure was used across the studies included, so instead treatment effects were transformed into standardized mean differences (SMDs) for NMA. Table A1 in the on‐line appendix A.12 lists the treatment codes and classes, and full results of the NMA are available in the accompanying R package.

#### 3.2.1 Contrast level analysis

Despite the complexity of the original analysis, a contrast level threshold analysis is straightforward. We consider the joint posterior distribution as if it arose from an NMA on 84 independent data points, each representing the aggregate direct evidence that is available on a single treatment contrast. Following the methods in Section 2.4, we construct an approximate hypothetical likelihood by using non‐negative least squares. The fitted hypothetical likelihood covariance matrix includes a single infinite variance for one contrast (treatment 7 *versus* 1), meaning that the direct evidence on this contrast is estimated to have no influence on the posterior distribution. The Kullback–Leibler divergence of the reconstructed posterior distribution from the true posterior distribution is 1.55, indicating that the hypothetical data are a reasonable approximation (interpreted as a log‐Bayes‐factor, greater than 1 but less than 3).

**Figure 8:**
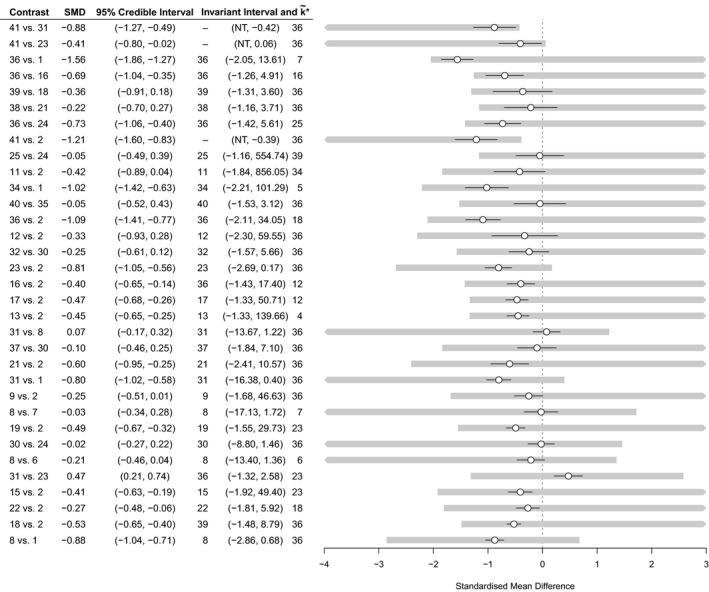
Contrast level forest plot for the social anxiety example showing results of the threshold analysis, sorted with smallest thresholds first (only contrasts with a threshold less than 2 SMDs are shown here for brevity; the complete results can be found in the Web supplementary material; the optimal treatment without bias adjustment is k*=41); NT, no threshold; ∘, SMD; —, 95% credible interval; 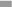, invariant interval

Owing to the large number of contrasts, Fig. 8 shows only the results of the threshold analysis for contrasts with thresholds of less than 2 SMDs. The optimal treatment under the original analysis is $k^{*}=41$, group cognitive behavioural therapy with phenelzine. No contrasts have invariant intervals which lie inside the 95% credible interval, meaning that the treatment recommendation is robust to the level of imprecision in the contrast level data. The smallest threshold is a positive change of 0.46 in the estimate of −0.88 SMD for the treatment 41 *versus* 31 contrast (the upper limit of the corresponding invariant interval is −0.88+0.46=−0.42), at which point treatment 36 (cognitive therapy) becomes optimal. Cohen (1988) considered an SMD of more than 0.8 to be large in the context of behavioural sciences; all except five thresholds are larger than this, and for each of these the new optimal treatment is treatment 36. Note that some invariant intervals are open on one side (indicated by ‘NT’) as there is no threshold in this direction; for these contrasts, a bias adjustment in this direction will never change the treatment decision. An important observation from this analysis is that the treatment recommendation is insensitive to changes in the combined evidence on the large majority of contrasts. Rather than performing a long and laborious qualitative assessment of all 84 contrasts and 100 studies, attention can be focused on the smaller number of contrasts (e.g. the five studies with thresholds smaller than 0.8 SMD) where plausible adjustments to the data may cause a change in treatment recommendation. Risk of bias assessments should be performed for these contrasts, and the thresholds and invariant intervals interpreted in light of the magnitude and direction of any potential bias.

#### 3.2.2 More complex analyses: pharmacological and psychological treatment bias

The methods that were described in Section 2 are easily extended to more complex decision rules and bias adjustment scenarios, simply by manipulating the set of $u_{ak^{⋆},m}$ values. Here, we have considered the effects of adjusting for a potential common bias (at the contrast level)among all pharmacological treatments (including combination therapies) and similarly among all psychological treatments. To do this, note that the influence of a common bias to a set of data points $\left\{y_{m}:m\in M\right\}$ (e.g. the pharmacological treatment contrasts *versus* inactive) is equal to the sum of the influences for each individual data point. Thus, the set of possible threshold solutions for a common bias is $\left\{u_{ak^{*},M}:a\in \left\{1,\ldots ,K\right\}\backslash k^{*}\right\}$, where

$$\begin{array}{cc} u_{ak^{*},M}= & \frac{-E(d_{ak^{*}})}{\sum_{m\in M}\left({\left[H\right]}_{k^{*}-1,m}-{\left[H\right]}_{a-1,m}\right)}\\ = & {\left(\sum_{m\in M}u_{ak^{*},m}^{-1}\right)}^{-1}. \end{array}$$

As before, the thresholds are then found by taking the smallest positive and negative solutions from this set.

**Figure 9:**
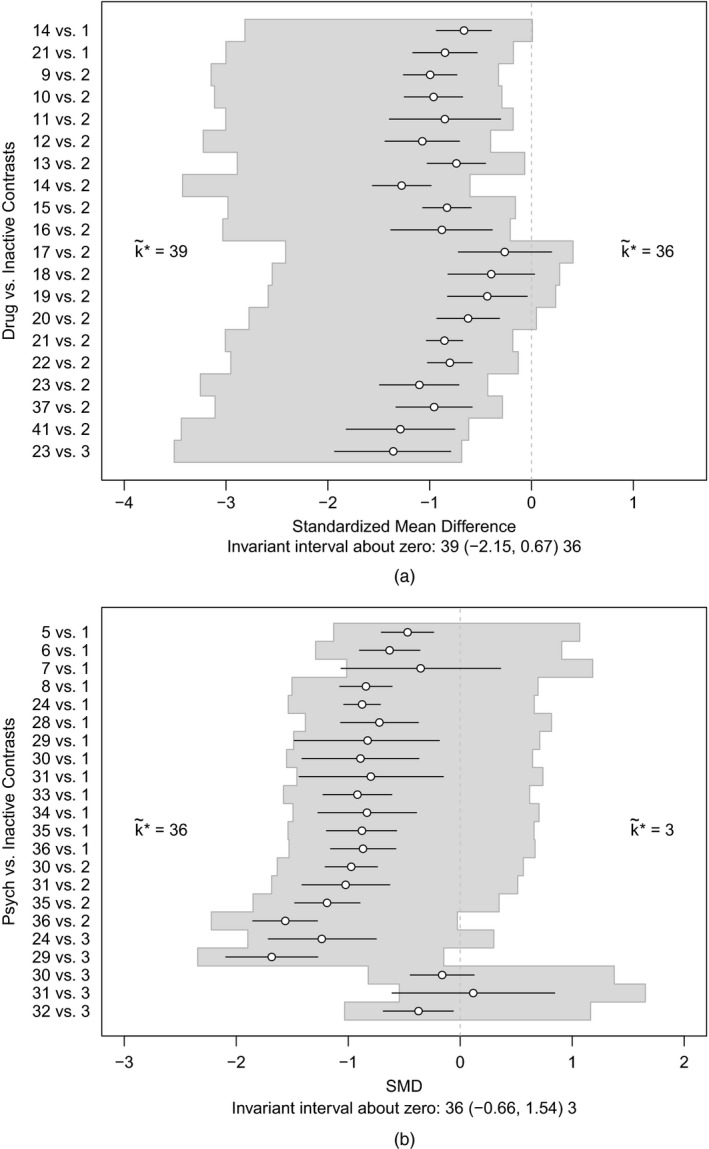
(a) Invariant interval for all pharmacological treatments against an inactive control, considered to be bias adjusted by the same amount, and (b) invariant interval for all psychological treatments against an inactive control, considered to be bias adjusted by the same amount; the optimal treatment without any bias adjustment is k*=41

**Figure 10:**
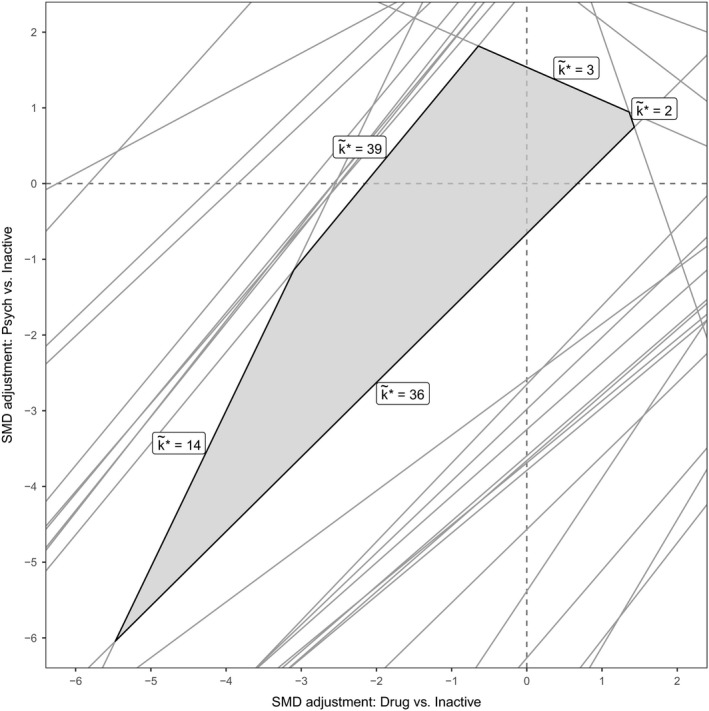
Invariant region (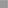) for simultaneous adjustments for common biases in all psychological and all pharmacological treatments: the new treatment recommendation at the boundary is shown as ${\tilde{k}}^{*}$; the optimal treatment without bias adjustment is *k* ^*^=41

The results of these analyses are shown in Fig. 9. In each case, at the smallest threshold, treatment 36 becomes optimal: with an adjustment of 0.67 SMD for all pharmacological treatments compared with inactive control (i.e. reducing their efficacy), or with an adjustment of −0.66 SMD for all psychological treatments compared with an inactive control (i.e. increasing their efficacy). The magnitude of these thresholds is large (Cohen, 1988)—probably much larger than any plausible common bias. We might also consider the effects of adjusting for these common biases simultaneously by examining the set of $u_{ak^{*},m}$ values. The resulting two‐dimensional invariant region is shown in Fig. 10. The size of the invariant region would probably reassure decision makers that adjustment for common pharmacological and/or psychological treatment effect biases (if they exist) would not affect the treatment recommendation.

## 4 Discussion

The threshold method that is presented in this paper enables researchers and decision makers to quantify the robustness of their conclusions to potentially biased evidence. Like all sensitivity analyses, it is useful for decision makers to know how robust their decision is to variations in assumptions and in the evidence inputs, especially if decisions are likely to be controversial, or when the quality of evidence is likely to be questioned. Current approaches based around the GRADE framework (Puhan *et al*., 2014; Salanti *et al*., 2014) give a thorough qualitative evaluation of the quality of evidence behind such decisions, but they fall short of describing the influence on treatment recommendations of any bias in the evidence. Providing bias adjustment thresholds and invariant regions can attest to the robustness of conclusions despite poor quality evidence, or it can highlight areas where the evidence should be carefully assessed for bias since bias adjustments of plausible magnitude could change the optimal treatment decision. Although our method gives quantitative results, their interpretation still requires qualitative judgements to determine which evidence might plausibly be biased and to what extent; the metaepidemiological literature on the empirical evidence for bias is likely to be helpful once potential biases have been identified in a risk of bias assessment (Savovic *et al*., 2012a). It is important to note that, although we highlight where a decision is sensitive to imprecision in the evidence when thresholds lie inside the CI or credible interval, it is entirely possible for larger bias adjustments to be plausible; for example, large studies yielding precise estimates may be biased beyond the range of their 95% CI. Although we have discussed only NMA of RCTs, the threshold method applies equally to analyses incorporating observational evidence; however, the potential for bias is much greater when including non‐randomized evidence, and the direction and magnitude of bias is difficult to predict.

It must be clear that threshold analyses do not seek to test for the presence or absence of bias; nor do they make any assumptions about the source, type or expected magnitude or direction of any bias. Rather, if any such bias was present, then subsequent adjustment would only alter the treatment decision if it were larger than the given thresholds. Knowledge of the likely nature of possible biases should be used in the planning and—most importantly—interpretation of threshold analyses.

Threshold analysis has previously been proposed by Caldwell *et al*. (2016), using a numerical method to derive thresholds based on a two‐stage Bayesian NMA. A particular feature of the method that is proposed in this paper is that it starts from the one‐stage Bayesian posterior distribution of relative treatment effects and manipulates it algebraically, rather than iteratively modifying the data. Not only can algebraic solutions be reached almost instantaneously by using matrix operations rather than lengthy and computationally expensive numerical techniques, but also this confers considerable flexibility; in practice, treatment recommendations are often based on complex models with multiple types of data input which would be difficult to fit into the two‐stage framework. Furthermore, the original data are not required for the threshold analysis to be performed, provided that posterior means and the covariance matrix of the parameters are available. Although not frequently published at present, this level of summary data is likely to be much easier to obtain on request than the full original data set; this significantly widens the scope of threshold analysis, compared with numerical methods.

An extension of this work is to embed the threshold method into a probabilistic CEA (Doubilet *et al*., 1985; Critchfield and Willard, 1986; Dias *et al*., 2013b), where the optimal treatment is found not by maximizing the posterior expected treatment effect as in equation (3), but by maximizing the posterior expected value of some net benefit function instead. A CEA seeks to weigh up the improvements in quality of life and life expectancy against the total costs for each treatment regimen, and this is achieved by the use of a net benefit function (Stinnett and Mullahy, 1998). Such analyses are used extensively by reimbursement agencies and threshold analysis would be useful to determine how bias adjustments can affect the outcome of a CEA. When the net benefit function is linear in treatment efficacy (or can be approximated as such), the threshold equations (6) can be easily transformed onto the net benefit scale. However, CEA models can be complex and often involve net benefit functions that are non‐linear; as such it would be useful to extend the threshold methodology to deal with non‐linear decision functions.

Other decision rules besides maximum efficacy or net benefit may be considered, e.g. recommending any active treatment if better than placebo, recommending a group of treatments whose efficacies are clinically equivalent (e.g. within some minimum clinically important difference or non‐inferiority margin) or restricting a recommendation to currently available treatments. More complex threshold analyses are also possible, e.g. to examine generic bias in a class of treatments or studies sharing given characteristics. All of these analyses are possible directly by examining the set of values *u*_*ab*,*m*_ (see equation (6)), giving the amount of adjustment to data point *y*_*m*_ which would see the posterior expectation of the contrast between treatments *a* and *b* change sign (so treatment preference between *a* and *b* switches).

Derivation of algebraic thresholds for the RE model is hindered by the analytic intractability of the joint posterior distribution when the between‐studies variance is given a prior distribution; instead we make the assumption that this variance is fixed and known so that conjugacy is preserved. This assumption should be tested by sensitivity analyses substituting plausible values of $\tau^{2}$, e.g. from the upper and lower limits of the 95% credible interval that is obtained from the NMA or from predictive distributions that are derived from similar meta‐analyses (Rhodes *et al*., 2015; Turner *et al*., 2015). There is empirical evidence that heterogeneity is greater in biased evidence bases (Savovic *et al*., 2012a,b), so it might be expected that $\tau^{2}$ would reduce after adjusting for bias (possibly beyond the lower credible limit).

Further applications of the threshold method are to metaregression and bias adjustment models (Dias *et al*., 2013b). The approach would follow from Section 2.3.4, where the additional parameters are regression covariates. Particular care should be taken to define an appropriate decision rule and in the interpretation of the treatment effect parameters since decisions can be different at different covariate values. The interpretation of the thresholds in this case is in terms of the adjustment for residual biases that are not accounted for in the model.

We have seen that the influence matrix **H** mapping the data **y** onto the posterior mean of the treatment effect parameters E(d) is central to the derivation of thresholds and describes how changes in individual data points affect the posterior means of the basic treatment effect parameters. The role of a related quantity, the hat or contributions matrix, has been highlighted by several researchers previously in the context of influence analysis (Konig *et al*., 2013; Krahn *et al*., 2013; Salanti *et al*., 2014). The hat matrix describes how changes in individual data points affect the predicted values (as opposed to the treatment parameters), and in the basic FE model is derived from the design and influence matrices as **XH**. Krahn *et al*. (2013) and Konig *et al*. (2013) used the hat matrix to visualize the ‘flow of evidence’ in an NMA and to analyse and detect inconsistency, which Salanti *et al*. (2014) utilized within the context of GRADE applied to NMA.

A key contribution of this paper has been the reconstruction of the influence matrix (and therefore the hat matrix) from the Bayesian posterior distribution. This allows considerable flexibility because, in practice, treatment recommendations may be based on complex models, e.g. including class effects, and may incorporate several types of data. The social anxiety guideline (National Collaborating Centre for Mental Health, 2013), for example, incorporates data on both response and recovery rates, and synthesizes trials reporting either ORs or outcomes on continuous scales. It is, of course, the need for flexible computation methods in the face of irregular and complex data that has made Bayesian Markov chain Monte Carlo sampling the method of choice in practical applications (Dias *et al*., 2013a).

Although we have derived results in a Bayesian context, the threshold method applies equally to NMAs that are performed within a frequentist framework. The influence matrix **H** can be written down for many frequentist estimation routines: for example, the maximum likelihood estimate for the treatment parameters in an FE model is of the form $\hat{d}={\left(X^{T}V^{-1}X\right)}^{-1}X^{T}V^{-1}y$, so the influence matrix is **H**=(**X** ^T^ **V** ^−1^ **X**)^−1^ **X** ^T^ **V** ^−1^, and we continue with equation (6) to derive thresholds as usual. In this case the result can also be reached by considering the frequentist framework as a special case of the Bayesian framework with the prior precision matrix $\Sigma_{d}^{-1}$ equal to 0; for other estimation routines the correspondence is not exact but may be a useful approximation if the influence matrix has no closed form.

One practical limitation of the threshold method is that there is no satisfactory way to display the results of simultaneous bias adjustments in more than three contrasts or data points. As we have shown in Section 2.5 the problem lies not with deriving bias adjustment thresholds in higher dimensions but in visualizing and interpreting them. We have given examples of how to visualize invariant regions in two dimensions (e.g. Fig. 6 in Section 3.1.3), and a similar approach is possible in three dimensions. For more than three contrasts or data points, a graphical representation of this kind is not possible, making interpretation difficult. However, in practice, if bias adjustment is to be considered for a large number of studies, it may be preferable to estimate the study level bias adjustment within the hierarchical NMA analysis, either by regression (Dias *et al*., 2010; Salanti *et al*., 2010; Naci *et al*., 2014) or by giving bias terms informative priors based on expert opinion (Turner *et al*., 2009; Welton *et al*., 2009; Dias *et al*., 2010). A potential avenue for future research into the effects of multiple simultaneous bias adjustments lies with the influence matrix. By examining this matrix it should be possible to identify whether bias adjustment in a given combination of data points may lead to wider invariant intervals, due to the influences of multiple data points partially cancelling out, or smaller invariant intervals, due to the combined influence increasing additively.

Importantly, threshold analysis of complex, hierarchical or otherwise atypical NMA models may always be performed at the contrast level, provided that the joint posterior distribution of the treatment effect parameters is available (either first hand from an analysis, or sufficiently reported in a published NMA), and that this joint posterior distribution is at least approximately normal. Under such conditions we can apply the methods that were proposed in Section 2.4 to derive bias adjustment thresholds and invariant intervals, regardless of the manner in which the joint posterior distribution arose. As such, the threshold method proposed is applicable to a wide range of situations that may be encountered by decision makers and has the potential to focus discussion on the risk of bias in particular studies or comparisons to which the final treatment recommendation is most sensitive.

## 5 Supplementary materials

### 5.1 Appendices

All appendices are contained in a separate on‐line document providing technical derivations, statements and proofs of theorems and lemmas, and notes on computation.

### 5.2 Computer code

An R package nmathresh is provided in the on‐line material that implements the threshold method, and the R code and data that were used to perform the example analyses are available from

http://wileyonlinelibrary.com/journal/rss-datasets

## Supporting information

‘Sensitivity of treatment recommendations to bias in network meta‐analysis: A, Appendices’.
