# Supplementary material for: Sensitivity of treatment recommendations to bias in network meta‐analysis
Source: J R Stat Soc Ser A Stat Soc. 2017 Dec 6;181(3):843–67. doi: 10.1111/rssa.12341 (PMC6221150; doi:10.1111/rssa.12341)
Supplement: Supplementary file 1 — ‘Sensitivity of treatment recommendations to bias in network meta‐analysis: A, Appendices’. [file RSSA-181-843-s001.pdf]

Sensitivity of treatment recommendations to bias in Network Meta-Analysis

David M Phillippo[1], Sofia Dias[1], AE Ades[1], Vanessa Didelez[2,3], Nicky J Welton[1]

[1]Population Health Sciences, Bristol Medical School, University of Bristol
[2]Leibniz Institute for Prevention Research and Epidemiology – BIPS
[3]Department of Mathematics, University of Bremen

A. APPENDICES

A.1 Lemma 1

Let $\tilde{k}^* = \underset{k=1,\ldots,K}{\operatorname{argmax}} \widetilde{\mathbb{E}}(d_k)$ be the optimal treatment under the bias-adjusted data $\tilde{\boldsymbol{y}}(\boldsymbol{\beta})$. Then:

a) $\tilde{k}^* \neq k^* \quad \Leftrightarrow \quad \widetilde{\mathbb{E}}\left(d_{bk^*}\right) < 0$ for at least one $b \in \{1,\ldots,K\} \setminus k^*$.

b) For a value of $\boldsymbol{\beta}$ where $\widetilde{\mathbb{E}}\left(d_{bk^*}\right) < 0$ for only one $b \in \{1,\ldots,K\} \setminus k^*$, the new optimal treatment is $\tilde{k}^* = b$.

Proof of Lemma 1a

Recall a general property of contrasts that $d_{ab} = -d_{ba}$. So without loss of generality, the set of contrasts associated with k^* is $\left\{d_{ak^*} : a \in \{1,\ldots,K\} \setminus k^*\right\}$. Note that under the original biased data the set of contrasts associated with k^* have posterior expected values satisfying $\mathbb{E}_{\boldsymbol{d}|\boldsymbol{y}}\left(d_{ak^*}\right) > 0 \quad \forall a \in \{1,\ldots,K\} \setminus k^*$.

First show that

$$\tilde{k}^* \neq k^* \quad \Rightarrow \quad \widetilde{\mathbb{E}}\left(d_{bk^*}\right) < 0 \text{ for at least one } b \in \{1,\ldots,K\} \setminus k^*.$$

We have that $\tilde{k}^*$ is the new optimal treatment under the bias-adjusted data, so by definition

$$\widetilde{\mathbb{E}}\left(d_{\tilde{k}^*}\right) > \widetilde{\mathbb{E}}\left(d_a\right) \quad \forall a \in \{1,\ldots,K\} \setminus \tilde{k}^*,$$

and in particular $\widetilde{\mathbb{E}}\left(d_{\tilde{k}^*}\right) > \widetilde{\mathbb{E}}\left(d_{k^*}\right)$. Thus

$$\widetilde{\mathbb{E}}\left(d_{\tilde{k}^*k^*}\right) = \widetilde{\mathbb{E}}\left(d_{k^*}\right) - \widetilde{\mathbb{E}}\left(d_{\tilde{k}^*}\right) < 0 .$$

That is, the posterior expectation of one of the contrasts associated with k^* has changed sign.

Now show that

$$\tilde{k}^* \neq k^* \quad \Leftarrow \quad \widetilde{\mathbb{E}}\left(d_{bk^*}\right) < 0 \text{ for at least one } b \in \{1,\ldots,K\} \setminus k^* .$$

As k^* is optimal under the original biased data, the posterior expectations of contrasts associated with k^* satisfy

$$\mathbb{E}_{\boldsymbol{d}|\boldsymbol{y}}\left(d_{ak^*}\right) = \mathbb{E}_{\boldsymbol{d}|\boldsymbol{y}}\left(d_{k^*}\right) - \mathbb{E}_{\boldsymbol{d}|\boldsymbol{y}}\left(d_a\right) > 0 \quad \forall a \in \{1,\ldots,K\} \setminus k^* ,$$

since $\mathbb{E}_{\boldsymbol{d}|\boldsymbol{y}}\left(d_{k^*}\right) = \max\limits_{a=1,\ldots,K} \mathbb{E}_{\boldsymbol{d}|\boldsymbol{y}}\left(d_a\right)$.

Now suppose we bias-adjust the data $\tilde{\boldsymbol{y}}(\boldsymbol{\beta})$ enough that at least one of the posterior expectations of the contrasts associated with k^* changes sign; the set $B = \left\{b : \widetilde{\mathbb{E}}\left(d_{bk^*}\right) < 0\right\} \subseteq \{1,\ldots,K-1\}$ is not empty.

We have then

$$\begin{aligned} \widetilde{\mathbb{E}}\left(d_{bk^*}\right) &= \widetilde{\mathbb{E}}\left(d_{k^*}\right) - \widetilde{\mathbb{E}}\left(d_b\right) < 0 \qquad \forall b \in B \\ \widetilde{\mathbb{E}}\left(d_{bk^*}\right) &= \widetilde{\mathbb{E}}\left(d_{k^*}\right) - \widetilde{\mathbb{E}}\left(d_b\right) \geq 0 \qquad \forall b \notin B \cup \left\{k^*\right\} \end{aligned}$$

Thus

$$\begin{aligned} \widetilde{\mathbb{E}}\left(d_{k^*}\right) &< \widetilde{\mathbb{E}}\left(d_b\right) \qquad \forall b \in B \\ \widetilde{\mathbb{E}}\left(d_{k^*}\right) &\geq \widetilde{\mathbb{E}}\left(d_b\right) \qquad \forall b \notin B \cup \left\{k^*\right\} \end{aligned}$$

And so choosing $\tilde{k}^* = \underset{b \in B}{\operatorname{argmax}}\, \widetilde{\mathbb{E}}\left(d_b\right)$ we have that

$$\widetilde{\mathbb{E}}\left(d_{\tilde{k}^*}\right) \geq \widetilde{\mathbb{E}}\left(d_b\right) > \widetilde{\mathbb{E}}\left(d_{k^*}\right) \geq \widetilde{\mathbb{E}}\left(d_{b'}\right) \qquad \forall \quad b \in B, \quad b' \notin B \cup \left\{k^*\right\} .$$

Therefore $\tilde{k}^*$ is the new optimal treatment and $\tilde{k}^* \neq k^*$ since $k^* \notin B$ and $\tilde{k}^* \in B$ by definition.

Proof of Lemma 1b

Let us choose a value of the bias adjustment $\boldsymbol{\beta}$ so that the posterior expectation of only one contrast changes sign, say $\widetilde{\mathbb{E}}\left(d_{bk^*}\right) < 0$.

Following on from the proof of part a) we see that $B = \{b\}$, a singleton set. Since $\tilde{k}^* \in B$ by definition, we therefore know that $\tilde{k}^* = b$.

A.2 Theorem: Posterior distribution for FE model

Given the following model with multivariate Normal likelihood and prior distribution

$$\begin{aligned} \boldsymbol{y} \mid \boldsymbol{d} &\sim \mathrm{N}\left(\boldsymbol{Xd}, \boldsymbol{V}\right) \\ \boldsymbol{d} &\sim \mathrm{N}\left(\boldsymbol{d}_0, \boldsymbol{\Sigma}_d\right) \end{aligned}$$

Then the prior distribution and likelihood are conjugate and the posterior distribution is also multivariate Normal

$$\boldsymbol{d} \mid \boldsymbol{y} \sim \mathrm{N}\left(\boldsymbol{\Sigma}_n\left(\boldsymbol{\Sigma}_d^{-1}\boldsymbol{d}_0 + \boldsymbol{X}^T\boldsymbol{V}^{-1}\boldsymbol{y}\right), \boldsymbol{\Sigma}_n\right),$$

where

$$\boldsymbol{\Sigma}_n^{-1} = \boldsymbol{\Sigma}_d^{-1} + \boldsymbol{X}^T\boldsymbol{V}^{-1}\boldsymbol{X}.$$

Proof

Using Bayes rule (Bayes 1763), the posterior distribution is

$$\begin{aligned} p\left(\boldsymbol{d} \mid \boldsymbol{y}\right) &\propto p\left(\boldsymbol{y} \mid \boldsymbol{d}\right) p\left(\boldsymbol{d}\right) \\ &\propto \exp\left(-\frac{1}{2}\left(\left(\boldsymbol{y} - \boldsymbol{Xd}\right)^T \boldsymbol{V}^{-1}\left(\boldsymbol{y} - \boldsymbol{Xd}\right) + \left(\boldsymbol{d} - \boldsymbol{d}_0\right)^T \boldsymbol{\Sigma}_d^{-1}\left(\boldsymbol{d} - \boldsymbol{d}_0\right)\right)\right) \\ &= \exp\left(-\frac{1}{2}\left(\boldsymbol{y}^T\boldsymbol{V}^{-1}\boldsymbol{y} - \boldsymbol{d}^T\boldsymbol{X}^T\boldsymbol{V}^{-1}\boldsymbol{y} - \boldsymbol{y}^T\boldsymbol{V}^{-1}\boldsymbol{Xd} + \boldsymbol{d}^T\boldsymbol{X}^T\boldsymbol{V}^{-1}\boldsymbol{Xd}\right.\right. \\ &\qquad \left.\left. + \boldsymbol{d}^T\boldsymbol{\Sigma}_d^{-1}\boldsymbol{d} - \boldsymbol{d}_0^T\boldsymbol{\Sigma}_d^{-1}\boldsymbol{d} - \boldsymbol{d}^T\boldsymbol{\Sigma}_d^{-1}\boldsymbol{d}_0 + \boldsymbol{d}_0^T\boldsymbol{\Sigma}_d^{-1}\boldsymbol{d}_0\right)\right) \end{aligned}$$

Then since $\boldsymbol{V}$ and $\boldsymbol{\Sigma}$ are symmetric, and noting proportionality with $\boldsymbol{d}$,

$$\propto \exp\left(-\frac{1}{2}\left(\boldsymbol{d}^T\left(\boldsymbol{\Sigma}_d^{-1}+\boldsymbol{X}^T\boldsymbol{V}^{-1}\boldsymbol{X}\right)\boldsymbol{d}-2\boldsymbol{d}^T\left(\boldsymbol{X}^T\boldsymbol{V}^{-1}\boldsymbol{y}+\boldsymbol{\Sigma}_d^{-1}\boldsymbol{d}_0\right)\right)\right)$$

And completing the square,

$$\propto \exp\left(-\frac{1}{2}\left(\boldsymbol{d}-\boldsymbol{d}_n\right)^T\boldsymbol{\Sigma}_n^{-1}\left(\boldsymbol{d}-\boldsymbol{d}_n\right)\right)$$

which we recognise as the multivariate Normal as required. See also Gelman *et al.* (2013, p. 71).

A.3 Derivation of bias adjustment thresholds for the basic FE model

We derive thresholds for the basic FE model described in section 2.3.2 by rearranging the expression for the posterior mean from equation (8). Under the bias adjusted data, this becomes

$$\begin{aligned}\widetilde{\mathbb{E}}(\boldsymbol{d})&=\boldsymbol{\Sigma}_n\left(\boldsymbol{\Sigma}_d^{-1}\boldsymbol{d}_0+\boldsymbol{X}^T\boldsymbol{V}^{-1}\left(\boldsymbol{y}+\boldsymbol{\beta}_m\right)\right)\\&=\mathbb{E}(\boldsymbol{d})+\boldsymbol{\Sigma}_n\boldsymbol{X}^T\boldsymbol{V}^{-1}\boldsymbol{\beta}_m\end{aligned} \tag{A.1}$$

Notice here that we can think of $\boldsymbol{\Sigma}_n\boldsymbol{X}^T\boldsymbol{V}^{-1}$ as an influence matrix, determining the change in the posterior mean resulting from a change in the data vector by $\boldsymbol{\beta}_m$. Let us denote this influence matrix as $\boldsymbol{H}$, and rewrite equation (A.1) as

$$\widetilde{\mathbb{E}}(\boldsymbol{d})=\mathbb{E}(\boldsymbol{d})+\boldsymbol{H}\boldsymbol{\beta}_m. \tag{A.2}$$

Consequently from Lemma 1 (appendix A.1), we must solve $K-1$ equations of the form

$$\widetilde{\mathbb{E}}\left(d_{ak^*}\right)=0,\quad \forall a\in\{1,\ldots,K\}\setminus k^* \tag{A.3}$$

to determine the point at which the expected value changes sign, and then take the smallest positive and negative solutions to be the positive and negative threshold values. Using (A.2) we can find solutions $u_{ak^*,m}$ to equation (A.3) expectation by rearranging the following equations:

$$\begin{aligned}0=\widetilde{\mathbb{E}}\left(d_{ak^*}\right)&=\widetilde{\mathbb{E}}\left(d_{k^*}\right)-\widetilde{\mathbb{E}}\left(d_a\right)\\&=\left[\widetilde{\mathbb{E}}(\boldsymbol{d})\right]_{k^*-1}-\left[\widetilde{\mathbb{E}}(\boldsymbol{d})\right]_{a-1}\\&=\mathbb{E}\left(d_{ak^*}\right)+\left([\boldsymbol{H}]_{k^*-1,m}-[\boldsymbol{H}]_{a-1,m}\right)u_{ak^*,m}\end{aligned} \tag{A.4}$$

for $a\in\{2,\ldots,K\}\setminus k^*$ when $k^*\neq 1$, and

$$
\begin{aligned}
0 = \widetilde{\mathbb{E}}\left(d_{1k^*}\right) &= \widetilde{\mathbb{E}}\left(d_{k^*}\right) \\
&= \mathbb{E}\left(d_{k^*}\right) + [\boldsymbol{H}]_{k^*-1,m}\, u_{1k^*,m}
\end{aligned} \tag{A.5}
$$

for $a = 1$ when $k^* \neq 1$. If $k^* = 1$ then the equations are of the form

$$
\begin{aligned}
0 = \widetilde{\mathbb{E}}\left(d_{1a}\right) &= \widetilde{\mathbb{E}}\left(d_{a}\right) \\
&= \mathbb{E}\left(d_{a}\right) + [\boldsymbol{H}]_{a-1,m}\, u_{1a,m}
\end{aligned} \tag{A.6}
$$

for $a \in \{2,\ldots,K\}$.

Now we simply re-arrange equations (A.4), (A.5), and (A.6) to arrive at the solutions:

For $k^* \neq 1$

$$
u_{ak^*,m} = \frac{-\mathbb{E}\left(d_{ak^*}\right)}{[\boldsymbol{H}]_{k^*-1,m} - [\boldsymbol{H}]_{a-1,m}}, \qquad \text{for } a \in \{2,\ldots,K\} \setminus k^*
$$

$$
u_{1k^*,m} = \frac{-\mathbb{E}\left(d_{1k^*}\right)}{[\boldsymbol{H}]_{k^*-1,m}}, \qquad \text{for } a = 1
$$

For $k^* = 1$

$$
u_{1a,m} = \frac{-\mathbb{E}\left(d_{1a}\right)}{[\boldsymbol{H}]_{a-1,m}}, \qquad \text{for } a \in \{2,\ldots,K\}
$$

The positive and negative threshold values $\beta_m^{+\text{thresh}}$ and $\beta_m^{-\text{thresh}}$ for a data point y_m are then

$$
\begin{aligned}
\beta_m^{+\text{thresh}} &= u_{bk^*,m} \text{ where } b = \underset{a \in \{1,\ldots,K\} \setminus k^*}{\operatorname{argmin}} \left\{ u_{ak^*,m} : u_{ak^*,m} > 0 \right\} \\
\beta_m^{-\text{thresh}} &= u_{bk^*,m} \text{ where } b = \underset{a \in \{1,\ldots,K\} \setminus k^*}{\operatorname{argmax}} \left\{ u_{ak^*,m} : u_{ak^*,m} < 0 \right\}
\end{aligned} \tag{A.7}
$$

Furthermore, from Lemma 1 (appendix A.1) and equation (A.7) we know that the new optimal treatment at the threshold is $\tilde{k}^* = b$. Note that, although it is in theory possible for there to be two or more treatment effects that are both exactly maximal at the threshold value of β, this happens with probability zero.

A.4 Theorem: Posterior distribution for RE model

Given the RE model

$$\text{Prior:} \quad \boldsymbol{d} \sim \mathrm{N}\left(\boldsymbol{d}_0, \boldsymbol{\Sigma}_d\right)$$
$$\text{Likelihood:} \quad \boldsymbol{y} \mid \boldsymbol{\delta} \sim \mathrm{N}\left(\boldsymbol{\delta}, \boldsymbol{V}\right)$$
$$\text{RE Model:} \quad \boldsymbol{\delta} \mid \boldsymbol{d} \sim \mathrm{N}\left(\boldsymbol{X}\boldsymbol{d}, \boldsymbol{\Sigma}_{\tau^2}\right)$$

where the between studies covariance matrix $\boldsymbol{\Sigma}_{\tau^2}$ is known and fixed, the joint posterior distribution for $\boldsymbol{d}$ and $\boldsymbol{\delta}$ is

$$\begin{pmatrix} \boldsymbol{d} \\ \boldsymbol{\delta} \end{pmatrix} \Bigg| \boldsymbol{y} \sim \mathrm{N}\left(\boldsymbol{\Sigma}_n \begin{pmatrix} \boldsymbol{\Sigma}_d^{-1}\boldsymbol{d}_0 \\ \boldsymbol{V}^{-1}\boldsymbol{y} \end{pmatrix}, \boldsymbol{\Sigma}_n \right)$$

where

$$\boldsymbol{\Sigma}_n = \begin{pmatrix} \boldsymbol{X}^T\boldsymbol{\Sigma}_{\tau^2}^{-1}\boldsymbol{X} + \boldsymbol{\Sigma}_d^{-1} & -\boldsymbol{X}^T\boldsymbol{\Sigma}_{\tau^2}^{-1} \\ -\boldsymbol{\Sigma}_{\tau^2}^{-1}\boldsymbol{X} & \boldsymbol{V}^{-1} + \boldsymbol{\Sigma}_{\tau^2}^{-1} \end{pmatrix}^{-1}.$$

Proof

Similarly to the proof for the posterior distribution of the FE model, the posterior distribution is

$$\begin{aligned} p(\boldsymbol{d},\boldsymbol{\delta} \mid \boldsymbol{y}) &\propto p(\boldsymbol{y} \mid \boldsymbol{\delta}) p(\boldsymbol{\delta} \mid \boldsymbol{d}) p(\boldsymbol{d}) \\ &\propto \exp\left\{ -\frac{1}{2}\left[(\boldsymbol{y}-\boldsymbol{\delta})^T \boldsymbol{V}^{-1} (\boldsymbol{y}-\boldsymbol{\delta}) + (\boldsymbol{\delta}-\boldsymbol{X}\boldsymbol{d})^T \boldsymbol{\Sigma}_{\tau^2}^{-1} (\boldsymbol{\delta}-\boldsymbol{X}\boldsymbol{d}) + (\boldsymbol{d}-\boldsymbol{d}_0)^T \boldsymbol{\Sigma}_d^{-1} (\boldsymbol{d}-\boldsymbol{d}_0) \right] \right\} \\ &\propto \exp\left\{ -\frac{1}{2}\left[\begin{pmatrix} \boldsymbol{d} \\ \boldsymbol{\delta} \end{pmatrix}^T \begin{pmatrix} \boldsymbol{X}^T\boldsymbol{\Sigma}_{\tau^2}^{-1}\boldsymbol{X} + \boldsymbol{\Sigma}_d^{-1} & -\boldsymbol{X}^T\boldsymbol{\Sigma}_{\tau^2}^{-1} \\ -\boldsymbol{\Sigma}_{\tau^2}^{-1}\boldsymbol{X} & \boldsymbol{V}^{-1} + \boldsymbol{\Sigma}_{\tau^2}^{-1} \end{pmatrix} \begin{pmatrix} \boldsymbol{d} \\ \boldsymbol{\delta} \end{pmatrix} - 2 \begin{pmatrix} \boldsymbol{d} \\ \boldsymbol{\delta} \end{pmatrix} \begin{pmatrix} \boldsymbol{\Sigma}_d^{-1}\boldsymbol{d}_0 \\ \boldsymbol{V}^{-1}\boldsymbol{y} \end{pmatrix} \right] \right\} \end{aligned}$$

which we recognise as the multivariate Normal as required. See also Gelman *et al.* (2013, p. 582).

A.5 Bias adjustment thresholds for the basic RE model

The joint posterior distribution of $\boldsymbol{d}$ and $\boldsymbol{\delta}$ (conditional on $\boldsymbol{\Sigma}_{\tau^2}$) is given in equation (9)(with proof in appendix A.4). We partition the joint covariance matrix as

$$\boldsymbol{\Sigma}_n = \begin{pmatrix} \boldsymbol{X}^T\boldsymbol{\Sigma}_{\tau^2}^{-1}\boldsymbol{X} + \boldsymbol{\Sigma}_d^{-1} & -\boldsymbol{X}^T\boldsymbol{\Sigma}_{\tau^2}^{-1} \\ -\boldsymbol{\Sigma}_{\tau^2}^{-1}\boldsymbol{X} & \boldsymbol{V}^{-1} + \boldsymbol{\Sigma}_{\tau^2}^{-1} \end{pmatrix}^{-1} = \begin{pmatrix} \boldsymbol{A}_* & \boldsymbol{B}_* \\ \boldsymbol{B}_*^T & \boldsymbol{C}_* \end{pmatrix}.$$

Under bias-adjusted data $\tilde{\boldsymbol{y}} = \boldsymbol{y} + \boldsymbol{\beta}_m$, the joint posterior mean of $\boldsymbol{d}$ and $\boldsymbol{\delta}$ becomes

$$\widetilde{\mathbb{E}}(\boldsymbol{d}) = \left[\boldsymbol{\Sigma}_n \begin{pmatrix} \boldsymbol{\Sigma}_d^{-1}\boldsymbol{d}_0 \\ \boldsymbol{V}^{-1}(\boldsymbol{y}+\boldsymbol{\beta}_m) \end{pmatrix} \right]_{1:K-1}$$
$$= \boldsymbol{A}_* \boldsymbol{\Sigma}_d^{-1}\boldsymbol{d}_0 + \boldsymbol{B}_*\boldsymbol{V}^{-1}\boldsymbol{y} + \boldsymbol{B}_*\boldsymbol{V}^{-1}\boldsymbol{\beta}_m$$
$$= \mathbb{E}(\boldsymbol{d}) + \boldsymbol{B}_*\boldsymbol{V}^{-1}\boldsymbol{\beta}_m$$

where we make use of a subscript colon notation to indicate subvectors (i.e. for a general vector $\boldsymbol{x}$ of length l, we define $\boldsymbol{x}_{p:q} = (x_p, \ldots, x_q)^T$ for $1 \leq p < q \leq l$). Then following the same arguments as the basic FE case (given in appendix A.3), the influence matrix is $\boldsymbol{H} = \boldsymbol{B}_*\boldsymbol{V}^{-1}$ and the thresholds are given by equations (5) and (6).

A.6 Bias adjustment thresholds for the extended FE model

The extended FE model with parameter vector $\boldsymbol{\gamma} = \begin{pmatrix} \boldsymbol{d} \\ \boldsymbol{\mu} \end{pmatrix}$ is written

$$\boldsymbol{y} \mid \boldsymbol{\gamma} \sim \mathrm{N}(\boldsymbol{X\gamma}, \boldsymbol{V})$$
$$\boldsymbol{\gamma} \sim \mathrm{N}(\boldsymbol{\gamma}_0, \boldsymbol{\Sigma}_\gamma)$$

with posterior distribution

$$\boldsymbol{\gamma} \mid \boldsymbol{y} \sim \mathrm{N}\left(\boldsymbol{\Sigma}_n\left(\boldsymbol{\Sigma}_\gamma^{-1}\boldsymbol{\gamma}_0 + \boldsymbol{X}^T\boldsymbol{V}^{-1}\boldsymbol{y}\right), \boldsymbol{\Sigma}_n\right),$$

where the posterior covariance matrix is $\boldsymbol{\Sigma}_n = \left(\boldsymbol{\Sigma}_\gamma^{-1} + \boldsymbol{X}^T\boldsymbol{V}^{-1}\boldsymbol{X}\right)^{-1}$.

Then the joint posterior expectation of the basic treatment effect parameters $\boldsymbol{d}$ under the bias-adjusted data is

$$\widetilde{\mathbb{E}}(\boldsymbol{d}) = \left[\boldsymbol{\Sigma}_n\left(\boldsymbol{\Sigma}_\gamma^{-1}\boldsymbol{\gamma}_0 + \boldsymbol{X}^T\boldsymbol{V}^{-1}(\boldsymbol{y}+\boldsymbol{\beta}_m)\right)\right]_{1:K-1}$$
$$= \mathbb{E}(\boldsymbol{d}) + \left[\boldsymbol{\Sigma}_n\boldsymbol{X}^T\boldsymbol{V}^{-1}\boldsymbol{\beta}_m\right]_{1:K-1}$$
$$= \mathbb{E}(\boldsymbol{d}) + \left[\boldsymbol{\Sigma}_n\boldsymbol{X}^T\boldsymbol{V}^{-1}\right]_{\text{rows } 1:K-1}\boldsymbol{\beta}_m$$

where we make use of a subscript colon notation to denote subvectors (as in appendix A.5) and similarly to subset rows of a matrix.

From here we continue exactly as in the basic case (appendix A.3) to derive bias adjustment thresholds using the threshold equations (5) and (6), where the influence matrix is now $\boldsymbol{H} = \left[\boldsymbol{\Sigma}_n \boldsymbol{X}^T \boldsymbol{V}^{-1}\right]_{\text{rows } 1:K-1}$.

A.7 Bias adjustment thresholds for the extended RE model

We extend the RE model to include additional parameters $\boldsymbol{\mu}$, which are given a Normal prior distribution, and have an associated design matrix $\boldsymbol{M}$. We also allow for random effects terms to only be included for certain data points, using a design matrix $\boldsymbol{L}$, for example in the case of absolute effect measure data where only non-reference arms have a random effect term (Dias *et al.* 2013a); in most other cases the matrix $\boldsymbol{L}$ is the identity matrix. The extended RE model is written as

$$
\begin{aligned}
&\text{Priors:} && \boldsymbol{d} \sim \mathrm{N}\left(\boldsymbol{d}_0, \boldsymbol{\Sigma}_d\right), \qquad \boldsymbol{\mu} \sim \mathrm{N}\left(\boldsymbol{\mu}_0, \boldsymbol{\Sigma}_\mu\right) \\
&\text{Likelihood:} && \boldsymbol{y} \mid \boldsymbol{\delta}, \boldsymbol{\mu} \sim \mathrm{N}\left(\boldsymbol{L\delta} + \boldsymbol{M\mu}, \boldsymbol{V}\right) \\
&\text{RE Model:} && \boldsymbol{\delta} \mid \boldsymbol{d}, \tau^2 \sim \mathrm{N}\left(\boldsymbol{Xd}, \boldsymbol{\Sigma}_{\tau^2}\right)
\end{aligned}
$$

The posterior distribution is then

$$
\left.\begin{pmatrix} \boldsymbol{d} \\ \boldsymbol{\delta} \\ \boldsymbol{\mu} \end{pmatrix}\right| \boldsymbol{y}, \tau^2 \sim \mathrm{N}\left(\boldsymbol{\Sigma}_n \begin{pmatrix} \boldsymbol{\Sigma}_d^{-1} \boldsymbol{d}_0 \\ \boldsymbol{L}^T \boldsymbol{V}^{-1} \boldsymbol{y} \\ \boldsymbol{\Sigma}_\mu^{-1} \boldsymbol{\mu}_0 + \boldsymbol{M}^T \boldsymbol{V}^{-1} \boldsymbol{y} \end{pmatrix}, \boldsymbol{\Sigma}_n \right)
$$

where

$$
\boldsymbol{\Sigma}_n = \begin{pmatrix} \boldsymbol{X}^T \boldsymbol{\Sigma}_{\tau^2}^{-1} \boldsymbol{X} + \boldsymbol{\Sigma}_d^{-1} & -\boldsymbol{X}^T \boldsymbol{\Sigma}_{\tau^2}^{-1} & \boldsymbol{0} \\ -\boldsymbol{\Sigma}_{\tau^2}^{-1} \boldsymbol{X} & \boldsymbol{\Sigma}_{\tau^2}^{-1} + \boldsymbol{L}^T \boldsymbol{V}^{-1} \boldsymbol{L} & -\boldsymbol{L}^T \boldsymbol{V}^{-1} \boldsymbol{M} \\ \boldsymbol{0} & -\boldsymbol{M}^T \boldsymbol{V}^{-1} \boldsymbol{L} & \boldsymbol{\Sigma}_\mu^{-1} + \boldsymbol{M}^T \boldsymbol{V}^{-1} \boldsymbol{M} \end{pmatrix}^{-1} =: \begin{pmatrix} \boldsymbol{A}_* & \boldsymbol{B}_* & \boldsymbol{D}_* \\ \boldsymbol{B}_*^T & \boldsymbol{C}_* & \boldsymbol{E}_* \\ \boldsymbol{D}_*^T & \boldsymbol{E}_*^T & \boldsymbol{F}_* \end{pmatrix} \qquad \text{(A.8)}
$$

noting that, as in the basic RE case, we can partition the posterior covariance matrix into blocks. Proof of the posterior distribution follows closely that of the basic RE model in appendix A.4.

The joint posterior expectation of the basic treatment effect parameters $\boldsymbol{d}$ under the bias-adjusted data $\tilde{\boldsymbol{y}}$ is then

$$\widetilde{\mathbb{E}}(\boldsymbol{d}) = \left[\boldsymbol{\Sigma}_n \begin{pmatrix} \boldsymbol{\Sigma}_d^{-1}\boldsymbol{d}_0 \\ \boldsymbol{L}^T\boldsymbol{V}^{-1}(\boldsymbol{y}+\boldsymbol{\beta}_m) \\ \boldsymbol{\Sigma}_\mu^{-1}\boldsymbol{\mu}_0 + \boldsymbol{M}^T\boldsymbol{V}^{-1}(\boldsymbol{y}+\boldsymbol{\beta}_m) \end{pmatrix} \right]_{1:K-1}$$

$$= \mathbb{E}(\boldsymbol{d}) + \left(\boldsymbol{B}_*\boldsymbol{L}^T + \boldsymbol{D}_*\boldsymbol{M}^T\right)\boldsymbol{V}^{-1}\boldsymbol{\beta}_m$$

Following the same arguments as before (appendix A.2), we see that the thresholds are given by equations (5) and (6) where the influence matrix is now $\boldsymbol{H} = \left(\boldsymbol{B}_*\boldsymbol{L}^T + \boldsymbol{D}_*\boldsymbol{M}^T\right)\boldsymbol{V}^{-1}$.

A.8 Bias adjustment thresholds for RE models including class effects

Further extending the RE model of section 2.3.4 to include class effects, we have

Priors:	$\boldsymbol{z} \sim \mathrm{N}(\boldsymbol{z}_0, \boldsymbol{\Sigma}_z), \quad \boldsymbol{\mu} \sim \mathrm{N}(\boldsymbol{\mu}_0, \boldsymbol{\Sigma}_\mu)$
Likelihood:	$\boldsymbol{y} \mid \boldsymbol{\delta}, \boldsymbol{\mu} \sim \mathrm{N}(\boldsymbol{L\delta} + \boldsymbol{M\mu}, \boldsymbol{V})$
RE Model:	$\boldsymbol{\delta} \mid \boldsymbol{d}, \tau^2 \sim \mathrm{N}(\boldsymbol{Xd}, \boldsymbol{\Sigma}_{\tau^2})$
Class Effects:	$\boldsymbol{d} \mid \boldsymbol{z} \sim \mathrm{N}(\boldsymbol{Zz}, \boldsymbol{\Sigma}_d)$

where $\boldsymbol{z}$ is the vector of class effect parameters and the matrix $\boldsymbol{Z}$ is a design matrix for the treatment clasfses. Each row of $\boldsymbol{Z}$ corresponds to a treatment, which is assigned a class by a 1 in the corresponding column and zeros elsewhere in the row. As before, we must work with the posterior distribution assuming that $\boldsymbol{\Sigma}_{\tau^2}$ is known, fixed, and invariant to bias adjustments; furthermore, we now assume that the between-treatment covariance matrix $\boldsymbol{\Sigma}_d$ is also known, fixed, and invariant to bias adjustments. Both of these assumptions can be tested using sensitivity analyses.

The posterior distribution is then

$$\left.\begin{pmatrix} \boldsymbol{d} \\ \boldsymbol{\delta} \\ \boldsymbol{\mu} \\ \boldsymbol{z} \end{pmatrix}\right| \boldsymbol{y}, \tau^2, \boldsymbol{\Sigma}_d \sim \mathrm{N}\left(\boldsymbol{\Sigma}_n \begin{pmatrix} \boldsymbol{0} \\ \boldsymbol{L}^T\boldsymbol{V}^{-1}\boldsymbol{y} \\ \boldsymbol{\Sigma}_\mu^{-1}\boldsymbol{\mu}_0 + \boldsymbol{M}^T\boldsymbol{V}^{-1}\boldsymbol{y} \\ \boldsymbol{\Sigma}_z^{-1}\boldsymbol{z}_0 \end{pmatrix}, \boldsymbol{\Sigma}_n \right)$$

where

$$\boldsymbol{\Sigma}_n = \begin{pmatrix} \boldsymbol{X}^T\boldsymbol{\Sigma}_{\tau^2}^{-1}\boldsymbol{X} + \boldsymbol{\Sigma}_d^{-1} & -\boldsymbol{X}^T\boldsymbol{\Sigma}_{\tau^2}^{-1} & \boldsymbol{0} & -\boldsymbol{\Sigma}_d^{-1}\boldsymbol{Z} \\ -\boldsymbol{\Sigma}_{\tau^2}^{-1}\boldsymbol{X} & \boldsymbol{\Sigma}_{\tau^2}^{-1} + \boldsymbol{L}^T\boldsymbol{V}^{-1}\boldsymbol{L} & -\boldsymbol{L}^T\boldsymbol{V}^{-1}\boldsymbol{M} & \boldsymbol{0} \\ \boldsymbol{0} & -\boldsymbol{M}^T\boldsymbol{V}^{-1}\boldsymbol{L} & \boldsymbol{\Sigma}_\mu^{-1} + \boldsymbol{M}^T\boldsymbol{V}^{-1}\boldsymbol{M} & \boldsymbol{0} \\ -\boldsymbol{Z}^T\boldsymbol{\Sigma}_d^{-1} & \boldsymbol{0} & \boldsymbol{0} & \boldsymbol{\Sigma}_d^{-1} + \boldsymbol{Z}^T\boldsymbol{\Sigma}_z^{-1}\boldsymbol{Z} \end{pmatrix}^{-1} =: \begin{pmatrix} \boldsymbol{A}_* & \boldsymbol{B}_* & \boldsymbol{D}_* & \boldsymbol{G}_* \\ \boldsymbol{B}_*^T & \boldsymbol{C}_* & \boldsymbol{E}_* & \boldsymbol{H}_* \\ \boldsymbol{D}_*^T & \boldsymbol{E}_*^T & \boldsymbol{F}_* & \boldsymbol{I}_* \\ \boldsymbol{G}_*^T & \boldsymbol{H}_*^T & \boldsymbol{I}_*^T & \boldsymbol{J}_* \end{pmatrix}$$

noting that, as in the basic RE case, we can partition the posterior covariance matrix into blocks. Proof of the posterior distribution follows closely that of the basic RE model in appendix A.4.

The joint posterior expectation of the basic treatment effect parameters $\boldsymbol{d}$ under the bias-adjusted data $\tilde{\boldsymbol{y}}$ is then

$$\begin{aligned} \widetilde{\mathbb{E}}(\boldsymbol{d}) &= \left[\boldsymbol{\Sigma}_n \begin{pmatrix} \boldsymbol{0} \\ \boldsymbol{L}^T\boldsymbol{V}^{-1}\boldsymbol{y} \\ \boldsymbol{\Sigma}_\mu^{-1}\boldsymbol{\mu}_0 + \boldsymbol{M}^T\boldsymbol{V}^{-1}\boldsymbol{y} \\ \boldsymbol{\Sigma}_z^{-1}\boldsymbol{z}_0 \end{pmatrix} \right]_{1:K-1} \\ &= \mathbb{E}(\boldsymbol{d}) + \left(\boldsymbol{B}_*\boldsymbol{L}^T + \boldsymbol{D}_*\boldsymbol{M}^T\right)\boldsymbol{V}^{-1}\boldsymbol{\beta}_m \end{aligned}$$

Following the same arguments as before (appendix A.3), we see that the thresholds are given by equations (5) and (6) where the influence matrix is now $H = \left(\boldsymbol{B}_*\boldsymbol{L}^T + \boldsymbol{D}_*\boldsymbol{M}^T\right)\boldsymbol{V}^{-1}$. Note that the influence matrix is identical to that in the extended RE case; we need do nothing different to the extended RE case despite the presence of class effects.

A.9 Forming an approximate dataset for contrast-level analysis

Suppose that we have the posterior summaries available from a one-stage Bayesian analysis for basic treatment effect parameters $\boldsymbol{d} = (d_2, \ldots, d_K)^T$, with joint posterior distribution given by $\boldsymbol{d} \mid \boldsymbol{y} \sim \mathrm{N}(\boldsymbol{\eta}, \boldsymbol{\Sigma})$. We wish to obtain a bias adjustment threshold for the combined data on each contrast d_{ab} for which there is direct evidence, $d_{ab} \in \mathcal{D}_{\text{dir}}$, where $\mathcal{D}_{\text{dir}}$ is the set of contrasts with direct evidence. We may think of the posterior distribution as arising from a NMA on $|\mathcal{D}_{\text{dir}}| \le K(K-1)/2$ data points y_{ab} with variances v_{ab}. These data points are hypothetical and are treated as independent of one another, each representing the combined evidence on a single contrast.

Assume a multivariate Normal likelihood for these hypothetical data points: $\boldsymbol{y} \mid \boldsymbol{d} \sim \mathrm{N}(\boldsymbol{Xd}, \boldsymbol{V})$ where $\boldsymbol{y} = (y_{ab} : d_{ab} \in \mathcal{D}_{\text{dir}})^T$, $\boldsymbol{V} = \mathrm{diag}(v_{ab} : d_{ab} \in \mathcal{D}_{\text{dir}})$, and $\boldsymbol{X}$ is a design matrix. Giving $\boldsymbol{d}$ the conjugate prior distribution $\boldsymbol{d} \sim \mathrm{N}(\boldsymbol{d}_0, \boldsymbol{\Sigma}_d)$ we have that the reconstructed posterior distribution based on the hypothetical data points is

$$\boldsymbol{d} \mid \boldsymbol{y} \sim \mathrm{N}\left(\left(\boldsymbol{\Sigma}_d^{-1} + \boldsymbol{X}^T\boldsymbol{V}^{-1}\boldsymbol{X}\right)^{-1}\left(\boldsymbol{\Sigma}_d^{-1}\boldsymbol{d}_0 + \boldsymbol{X}^T\boldsymbol{V}^{-1}\boldsymbol{y}\right), \left(\boldsymbol{\Sigma}_d^{-1} + \boldsymbol{X}^T\boldsymbol{V}^{-1}\boldsymbol{X}\right)^{-1}\right).$$

Equating this with the true joint posterior distribution $\mathrm{N}(\boldsymbol{\eta}, \boldsymbol{\Sigma})$ reported by the original NMA, we see that $\boldsymbol{\eta} = \left(\boldsymbol{\Sigma}_d^{-1} + \boldsymbol{X}^T\boldsymbol{V}^{-1}\boldsymbol{X}\right)^{-1}\left(\boldsymbol{\Sigma}_d^{-1}\boldsymbol{d}_0 + \boldsymbol{X}^T\boldsymbol{V}^{-1}\boldsymbol{y}\right)^{-1}$ and

$$\boldsymbol{\Sigma} = \left(\boldsymbol{\Sigma}_d^{-1} + \boldsymbol{X}^T\boldsymbol{V}^{-1}\boldsymbol{X}\right)^{-1}. \tag{A.9}$$

In order to calculate bias adjustment thresholds using the results of section 2.3.2, we require the influence matrix $\boldsymbol{H} = \boldsymbol{\Sigma X}^T\boldsymbol{V}^{-1}$. However, we cannot immediately evaluate $\boldsymbol{H}$ as the hypothetical likelihood precision matrix $\boldsymbol{V}^{-1}$ is required. Instead, exploiting the structure of the design matrix $\boldsymbol{X}$, we obtain $\boldsymbol{V}^{-1}$ (or an approximation) using equation (A.9) which we then use to evaluate the influence matrix and derive thresholds.

To obtain $\boldsymbol{V}^{-1}$, firstly, note that there are $|\mathcal{D}_{\text{dir}}|$ unknowns in $\boldsymbol{V}^{-1}$, as this matrix is diagonal with elements $p_{ab} = v_{ab}^{-1}$ by assumption. Secondly, note that $\boldsymbol{X}$ is structured with a row for each data point y_{ab}, with a 1 in column $b-1$ and, provided $k > 1$, with a –1 in column $a-1$; this structure results in the symmetric matrix

$$\boldsymbol{X}^T\boldsymbol{V}^{-1}\boldsymbol{X} = \begin{pmatrix} p_{12} + \sum_{b=3}^{K} p_{2b} & -p_{23} & -p_{24} & \cdots & -p_{2K} \\ & p_{13} + p_{23} + \sum_{b=4}^{K} p_{3b} & -p_{34} & \cdots & -p_{3K} \\ & & p_{14} + \sum_{a=2}^{3} p_{a4} + \sum_{b=5}^{K} p_{4b} & \ddots & \vdots \\ & & & \ddots & -p_{(K-1)K} \\ & & & & p_{1K} + \sum_{a=2}^{K-1} p_{aK} \end{pmatrix}$$

where $p_{ab} = 0$ if $d_{ab} \notin \mathcal{D}_{\text{dir}}$. We then rearrange equation (A.9) to equate $\boldsymbol{X}^T \boldsymbol{V}^{-1} \boldsymbol{X} = \boldsymbol{\Sigma}^{-1} - \boldsymbol{\Sigma}_d^{-1}$, resulting in $K(K-1)/2$ equations to solve in $|\mathcal{D}_{\text{dir}}|$ unknowns as the matrices are symmetric.

When the evidence network is complete $|\mathcal{D}_{\text{dir}}| = K(K-1)/2$ and the number of equations equals the number of unknowns. We therefore easily find a unique solution for each p_{ab}.

When there are some treatments with no direct evidence comparing them $|\mathcal{D}_{\text{dir}}| < K(K-1)/2$ and we have fewer unknowns than equations; the system of equations is overdetermined. We proceed to approximate $\boldsymbol{V}^{-1}$ using non-negative least squares (Lawson and Hanson 1995).

To examine how well the hypothetical likelihood recreates the posterior distribution we suggest evaluating the Kullback-Leibler (KL) divergence (Kullback and Leibler 1951) of the reconstructed posterior distribution with covariance matrix $\hat{\boldsymbol{\Sigma}} := \left(\boldsymbol{\Sigma}_d^{-1} + \boldsymbol{X}^T \boldsymbol{V}^{-1} \boldsymbol{X}\right)^{-1}$ from the true posterior distribution with covariance matrix $\boldsymbol{\Sigma}$; the KL divergence between two multivariate Normal distributions with the same mean is given by

$$\frac{1}{2}\left(\log \frac{|\hat{\boldsymbol{\Sigma}}|}{|\boldsymbol{\Sigma}|} + \operatorname{tr}\left(\hat{\boldsymbol{\Sigma}}^{-1}\boldsymbol{\Sigma}\right) - (K-1) \right).$$

The KL divergence is always non-negative and smaller values are desirable, indicating that the approximation reconstructs the posterior distribution well. Noting that the KL divergence is equivalent to the expected value of a log Bayes factor, we refer to Kass and Raftery (1995) for interpretation: for example, a KL divergence less than 1 is negligible, and values greater than 3 may be considered large. If evaluation of the KL divergence suggests a bad approximation, the contrast-level threshold analysis may give inaccurate results.

Once the hypothetical likelihood covariance matrix has been reconstructed, the thresholds are then evaluated as before using equations (5) and (6) with the influence matrix $\boldsymbol{H} = \boldsymbol{\Sigma} \boldsymbol{X}^T \boldsymbol{V}^{-1}$. Note that we cannot re-evaluate the posterior means under the bias-adjusted data to obtain $\tilde{k}^*$ as we do not have the hypothetical data, but we can use the result of Lemma 1 (appendix A.1) to efficiently obtain the new optimal treatment.

A.10 Computation

The threshold methods described in sections 2.3, 2.4, and 2.5 are implemented in R (version 3.0.1 or later) (R Core Team 2016), where we take advantage of the computational efficiency of vector and matrix operations to derive every threshold at the same time instead of looping over all data points and all treatments. In section A.10.1 we briefly demonstrate how such an approach is formulated mathematically. The resulting computation times are almost instantaneous; the examples presented in section 3 of this paper each took less than 0.01 seconds to calculate thresholds on a standard desktop PC. An R package *nmathresh* is provided in the supplementary material, containing all of the functions described below plus the data required for the examples in section 3. The package also contains a vignette which details the exact commands used for the examples.

The R function *nma_thresh* implements the study-level threshold method for FE models (sections 2.3.2 and 2.3.4) and RE models (sections 2.3.3, 2.3.4, and 2.3.5), depending on the value of the *nmatype* parameter (either *"fixed"* or *"random"* respectively). This function takes the posterior mean of the relative treatment effects, the likelihood and posterior covariance matrices, and the design matrix/matrices as inputs. The R function *nma_thresh* is also used to perform contrast-level analysis (section 2.4). In this scenario the hypothetical likelihood covariance matrix is constructed from the prior and posterior covariance matrices and the design matrix using the R function *recon_vcov*, either exactly or using non-negative least squares (NNLS) (Lawson and Hanson 1995) via the function *nnls* from the package *nnls* (Mullen and van Stokkum 2012). The R function *thresh_forest* takes the results from the NMA and threshold analysis and presents them graphically on a forest plot.

A.10.1 Mathematical derivation

Here we briefly describe mathematically the derivation of bias adjustment thresholds in a vectorised manner, allowing for highly efficient computation that does not rely on looping.

First, define a matrix $\boldsymbol{U}$ which contains the elements $u_{ak^*,m}$:

$$
\boldsymbol{U}=\begin{pmatrix} u_{1k^*,1} & \cdots & u_{1k^*,N} \\ \vdots & & \vdots \\ u_{(k^*-1)k^*,1} & \cdots & u_{(k^*-1)k^*,N} \\ u_{(k^*+1)k^*,1} & \cdots & u_{(k^*+1)k^*,N} \\ \vdots & & \vdots \\ u_{Kk^*,1} & \cdots & u_{Kk^*,N} \end{pmatrix}
$$

where each row corresponds to a contrast d_{ak^*} and each column to a data point y_m.

Recall that each $u_{ak^*,m}$ is of the form $u_{ak^*,m} = -\mathbb{E}\left(d_{ak^*}\right) / \left([\boldsymbol{H}]_{k^*-1,m} - [\boldsymbol{H}]_{a-1,m}\right)$ where $\boldsymbol{H}$ is the influence matrix of the data $\boldsymbol{y}$ on the joint posterior mean of $\boldsymbol{d}$, and so each $u_{ak^*,m}$ consists of a numerator and denominator. Next construct the matrix $\boldsymbol{D}$ which describes the linear combinations of elements of the influence matrix found in the denominator of each $u_{ak^*,m}$ element of $\boldsymbol{B}$:

$$
\boldsymbol{D}=\begin{pmatrix} 0 & \cdots & 0 & 1 & 0 & \cdots & \cdots & 0 \\ -1 & \ddots & \vdots & 1 & \vdots & & & \vdots \\ 0 & \ddots & 0 & \vdots & \vdots & & & \vdots \\ \vdots & \ddots & -1 & 1 & 0 & & & \vdots \\ \vdots & & 0 & 1 & -1 & \ddots & & \vdots \\ \vdots & & \vdots & \vdots & 0 & \ddots & \ddots & \vdots \\ \vdots & & \vdots & \vdots & \vdots & \ddots & \ddots & 0 \\ 0 & \cdots & 0 & 1 & 0 & \cdots & 0 & -1 \end{pmatrix}
$$

where the $\left(k^*-1\right)$-th column of $\boldsymbol{D}$ is filled with 1s. We can now write the matrix $\boldsymbol{U}$ using the formula

$$
\boldsymbol{U} = \left[-\operatorname{diag}\left(\mathbb{E}\left(d_{ak^*}\right) : a \in \{1,\ldots,K\} \setminus k^*\right)\right]\left[1/(\boldsymbol{DH})\right] \qquad \text{(A.10)}
$$

where $\operatorname{diag}\left(\mathbb{E}\left(d_{ak^*}\right) : a \in \{1,\ldots,K\} \setminus k^*\right)$ denotes the diagonal matrix with elements being the posterior expected values of contrasts associated with k^*, and $1/(\boldsymbol{DH})$ denotes division performed elementwise, that is $\left[1/(\boldsymbol{DH})\right]_{i,j} = 1/[\boldsymbol{DH}]_{i,j}$. Threshold values and invariant intervals for each data point y_m are then simply found by examining the m-th column of $\boldsymbol{U}$ from equation (A.10).

A.11 Thrombolytics Example: Formulating the NNLS problem for the contrast-level case

The design matrix for the hypothetical data is

$$\boldsymbol{X} = \begin{pmatrix} 1 & 0 & 0 & 0 & 0 \\ 0 & 1 & 0 & 0 & 0 \\ 0 & 0 & 1 & 0 & 0 \\ 0 & 0 & 0 & 1 & 0 \\ 0 & -1 & 1 & 0 & 0 \\ 0 & -1 & 0 & 1 & 0 \\ 0 & -1 & 0 & 0 & 1 \end{pmatrix}.$$

We need now to reconstruct the hypothetical likelihood covariance matrix $\boldsymbol{V}$. Letting the likelihood precision matrix $\boldsymbol{V}^{-1}$ have diagonal elements $p_{12}, p_{13}, p_{14}, p_{15}, p_{34}, p_{35}, p_{36}$, we see that

$$\boldsymbol{X}^T\boldsymbol{V}^{-1}\boldsymbol{X} = \begin{pmatrix} p_{12} & 0 & 0 & 0 & 0 \\ & p_{13}+p_{34}+p_{35}+p_{36} & -p_{34} & -p_{35} & -p_{36} \\ & & p_{14}+p_{34} & 0 & 0 \\ & & & p_{15}+p_{35} & 0 \\ & & & & p_{36} \end{pmatrix} \tag{A.11}$$

then using the fact that $\boldsymbol{\Sigma}_n^{-1} = \boldsymbol{\Sigma}_d^{-1} + \boldsymbol{X}^T\boldsymbol{V}^{-1}\boldsymbol{X}$ we equate (A.11) with

$$\boldsymbol{\Sigma}_n^{-1} - \boldsymbol{\Sigma}_d^{-1} = \begin{pmatrix} q_{22} & q_{23} & q_{24} & q_{25} & q_{26} \\ & q_{33} & q_{34} & q_{35} & q_{36} \\ & & q_{44} & q_{45} & q_{46} \\ & & & q_{55} & q_{56} \\ & & & & q_{66} \end{pmatrix} \tag{A.12}$$

Thus we have a system of $K(K-1)/2 = 15$ equations with $\text{length}(\boldsymbol{y}) = 7$ unknowns to solve; an overdetermined system. We can write the system of equations (A.11) and (A.12) in the form $R\boldsymbol{p} = \boldsymbol{q}$, where

$$\boldsymbol{p} = (p_{12}, p_{13}, p_{14}, p_{15}, p_{34}, p_{35}, p_{36})^T$$
$$\boldsymbol{q} = (q_{12}, q_{13}, q_{14}, q_{15}, q_{16}, q_{23}, q_{24}, q_{25}, q_{26}, q_{34}, q_{35}, q_{36}, q_{45}, q_{46}, q_{56})^T$$

and

$$
\boldsymbol{R} = \begin{pmatrix}
1 & 0 & 0 & 0 & 0 & 0 & 0 \\
0 & 0 & 0 & 0 & 0 & 0 & 0 \\
0 & 0 & 0 & 0 & 0 & 0 & 0 \\
0 & 0 & 0 & 0 & 0 & 0 & 0 \\
0 & 0 & 0 & 0 & 0 & 0 & 0 \\
0 & 1 & 0 & 0 & 1 & 1 & 1 \\
0 & 0 & 0 & 0 & -1 & 0 & 0 \\
0 & 0 & 0 & 0 & 0 & -1 & 0 \\
0 & 0 & 0 & 0 & 0 & 0 & -1 \\
0 & 0 & 1 & 0 & 1 & 0 & 0 \\
0 & 0 & 0 & 0 & 0 & 0 & 0 \\
0 & 0 & 0 & 0 & 0 & 0 & 0 \\
0 & 0 & 0 & 1 & 0 & 1 & 0 \\
0 & 0 & 0 & 0 & 0 & 0 & 0 \\
0 & 0 & 0 & 0 & 0 & 0 & 1
\end{pmatrix}.
$$

The NNLS problem is then

$$
\begin{aligned}
&\text{Minimise} && \|\boldsymbol{Rp}-\boldsymbol{q}\|^2 \\
&\text{Subject to} && \boldsymbol{p} \geq 0
\end{aligned}
$$

A.12 Social Anxiety Example: Treatment Codes

Table A1: Social Anxiety treatment codes and classes.

Code	Treatment	Class
1	Wait List	Wait List
2	Pill Placebo	Pill Placebo
3	Psychological Placebo	Psychological Placebo
4	Exercise Promotion	Exercise
5	Book	Self-help without support
6	Internet	
7	Book	Self-help with support
8	Internet	
9	Pregabalin	Anticonvulsants
10	Levetiracetam	
11	Gabapentin	
12	Mirtazapine	Noradrenaline and selective serotonin antagonists
13	Sertraline	SSRIs
14	Citalopram	
15	Escitalopram	
16	Fluoxetine	
17	Fluvoxamine	
18	Paroxetine	

19	Venlafaxine	
20	Alprazolam	Benzodiazepines
21	Clonazepam	
22	Moclobemide	MAOIs
23	Phenelzine	
24	Exposure in vivo	Exposure in vivo and social skills training
25	Social skills training	
26	Supportive therapy	Other psychological interventions
27	Mindfullness	
28	Interpersonal	
29	Short-term psychodynamic	Short-term psychodynamic
30	CBT	CBT - Group
31	CBT (Heimberg)	
32	CBT (Enhanced)	
33	CT (Shortened)	CBT - Individual
34	CBT (Heimberg)	
35	CBT	
36	CT	
37	Group CBT with fluoxetine	Combined psychological and drug
38	Psychodynamic with clonazepam	
39	Paroxetine with clonazepam	
40	Group CBT with moclobemide	
41	Group CBT with phenelzine	
